# Unraveling Image and Justice Concerns: A Social Identity Account on Appraisals and Emotional Drivers of High-Status Transgressor Group Members’ Solidarity With Low-Status Groups

**DOI:** 10.1177/01461672241252871

**Published:** 2024-06-18

**Authors:** Hakan Çakmak, Ernestine H. Gordijn, Yasin Koc, Katherine E. Stroebe

**Affiliations:** 1 University of Groningen, The Netherlands

**Keywords:** solidarity-based collective action, intergroup transgression, group identification, appraisal, emotion

## Abstract

High-status group members typically respond defensively when their ingroup members transgress against low-status groups. However, when they identify highly with transgressor groups, they sometimes also engage in solidarity with victimized low-status groups due to ingroup-focused motives. Yet, the response of low-identified transgressor group members, who can prioritize victims’ plight over ingroup interests, remains underexplored. To address this gap, we conducted three preregistered studies (*N_total_* = 886) concerning education-based transgressions in the Netherlands and the United Kingdom, employing cross-sectional (Study 1) and experimental designs (Studies 2–3). Supporting previous research, we found that high-identifiers engage in nonradical solidarity driven by ingroup image concerns and image-related emotions. Low-identifiers, however, engage in both nonradical and radical solidarity through perceived injustice and justice-related emotions. Our findings provide insights into the roots of high-status group collective action on behalf of low-status groups against intergroup transgressions. Theoretical and societal implications were discussed.

Low-status or socially disadvantaged groups oftentimes face transgressions, including discrimination, prejudice, exploitation, or exclusion based on factors like ethnicity, gender, socioeconomic status, or other aspects of social identity. When these transgressions are committed by high-status or socially advantaged group members, they are termed high-status group transgressions (Shuman et al., 2018). How do other high-status group members respond when their ingroup members are involved in such transgressions? Despite often reacting defensively (e.g., Gunn & Wilson, 2011), high-status group members sometimes express solidarity with victimized outgroups, for instance, through collective actions (i.e., collaborative efforts of individuals or groups rallying to address a common issue; Agostini & van Zomeren, 2021; van Zomeren et al., 2008). Solidarity refers to high-status group members participating in collective actions to address low-status groups’ sufferings (Radke et al., 2020). This solidarity becomes particularly prominent when transgressions are evident (e.g., Iyer et al., 2007; Shuman et al., 2018).

Examining the roots of this solidarity, previous work by Shuman et al. (2018) emphasized the role of social identification (i.e., one’s sense of belonging, commitment, and satisfaction within a social group; Tajfel, 1974). Research indicates that when transgressor group members experience group-based image concerns and emotions like guilt and shame, they tend to show solidarity with victims (Iyer et al., 2007; Shuman et al., 2018). This inclination is stronger when individuals strongly identify with their ingroups (Shuman et al., 2018). However, this image-based solidarity route focuses primarily on ingroup-oriented responses, addressing how high-status group members manage the consequences of transgressions *for their own group’s interests* (e.g., van Leeuwen & Täuber, 2012). Given the unfair victimization of low-status outgroups resulting from these transgressions, an alternative psychological process may exist, whereby some high-status group members are concerned about *the victimized outgroups’ needs*. To our knowledge, this alternative route to solidarity taken by high-status transgressors toward victimized outgroups remains unexplored, aside from some theoretical propositions (e.g., Radke et al., 2020).

To bridge this gap, we propose a justice-based pathway to solidarity that runs alongside the image-based route. This pathway is primarily influenced by the recognition of the unjust nature of ingroup transgressions (Lerner, 2003). We further suggest that, especially when justice-related emotions like anger and outrage accompany perceived outgroup injustice, this pathway may lead high-status group members to express solidarity distinct from those associated with the image-based route. The unique feature of this solidarity pathway is its prioritization of victimized outgroups’ needs over ingroup interests. We argue that this outgroup-oriented solidarity pathway becomes more pronounced when high-status group members have weaker ingroup attachments (i.e., low identification). Consequently, we contend that, in cases of ingroup transgressions, low-identifiers may follow the justice-based solidarity route *more than high-identifiers*, whereas high-identifiers could be *more inclined than low-identifiers* toward the image-based solidarity route.

## The Roots of the High-Identifiers’ Solidarity With Victimized Low-Status Groups

High-status group memberships, compared to low-status ones, often bring psychological benefits, such as positive well-being (Schmitt et al., 2014; cf. Hinton et al., 2022) and material advantages, including better access to monetary gains (Knowles & Peng, 2005). These groups are not only personally rewarding but are also perceived positively, even by members of other groups (Czopp et al., 2015). Consequently, high-status group members enjoy a favorable public image. Specifically, those who strongly identify with these groups reap these benefits and recognize this positive ingroup public image (Leach et al., 2008). However, transgressive behaviors by some ingroup members toward outgroups undermine their group’s positive public image, at least in the eyes of others (Otten & Gordijn, 2014; Shuman et al., 2018). When witnessing such cases, high-identifiers might become more concerned about their group’s image than low-identifiers (Otten & Gordijn, 2014) and may show helping and solidarity intentions to restore their group’s reputation (Shuman et al., 2018; van Leeuwen & Täuber, 2012).

Nonetheless, high-identifiers’ group-based image concerns per se can either positively or negatively impact outgroup solidarity (Wenzel et al., 2020). Concerns about group image were shown to drive defensive responses to ingroup transgressions, including justifications or downplaying the outgroups’ suffering (Bilali & Vollhardt, 2019; Gunn & Wilson, 2011). The key factor at play in dampening defensiveness and fostering solidarity with victimized outgroups appears to be the coupling role of image-related emotions (Gunn & Wilson, 2011). This is because image-related emotions like guilt and shame are linked to recognizing the ingroup’s responsibility for harming outgroups or being associated with transgressors and the loss of positive ingroup idealization (Doosje et al., 1998). Relatedly, Shuman et al. (2018) investigated the solidarity intentions of American nationals toward war prisoners tortured by U.S. troops in the Guantanamo Detention Camp. They found that increased group-based image concerns resulting from misconduct predicted solidarity for victims among individuals with a stronger American national identification, but only when it was associated with group-based guilt. Hence, image-related appraisals and emotions seem crucial for high-identifiers’ solidarity with victimized outgroups.

Another image-related emotion that is associated with outgroup solidarity is shame (see Hakim et al., 2021). Yet, prior studies showed distinct associations of shame and guilt with different outgroup solidarity forms. For example, Brown et al. (2008) demonstrated that group-based guilt was a stronger predictor than shame for transgressive group members’ reparatory attitudes toward victims in the context of colonial injustice. Yet, Gunn and Wilson (2011) provided evidence that both guilt and shame predicted reparation intentions, such as compensations, in gendered and colonial injustice contexts. Moreover, Iyer et al. (2007) found that shame felt by British and American citizens toward their countries’ invasion of Iraq predicted withdrawal attitudes, while guilt did not. These seemingly contradictory findings can be reconciled by considering that these studies examined more specific outcome variables within the broader concept of solidarity. Also, previous research often treated the shared variance between these emotions as measurement errors, investigating each emotion’s *unique effects* by controlling for the overlap between the two. However, such an overlap warrants closer attention and may indicate construct similarity (see Cameron et al., 2015, p. 382; Gordijn et al., 2006, p. 28), which is why we conceptualized them together as “image-related emotions.” We thus suggest that image-related emotions toward ingroup transgressions may predict high-status transgressor group members’ solidarity intentions with victims when measuring solidarity in generic terms. Consequently, group-based image concerns, which could lead to ingroup defensive strategies (Gunn & Wilson, 2011), could also lead to strategies to constructively manage ingroup-image concerns through increased feelings such as guilt and shame. We thus propose that, relative to low-identifiers, group-based image concerns and image-related emotions are the roots of high-identifiers’ solidarity with victimized low-status groups.

## The Roots of the Low-Identifiers’ Solidarity With Victimized Low-Status Groups

Low-identifiers are less concerned than high-identifiers with preserving the positive ingroup image (Teixeira et al., 2023). We argue that this holds true even when observing ingroup wrongdoings toward other groups. At the same time, it seems that low-identifiers are more likely than high-identifiers in taking the perspective of victimized outgroups (Radke et al., 2020; Zebel et al., 2009). Therefore, we assume that low-identifiers are more likely to recognize the transgressions against the outgroup as unjust, and these justice, rather than image, concerns may drive their outgroup solidarity.

Support for this assumption comes from the just-world beliefs literature. As Lerner and Miller (1978) stated, “As events become closer to their world . . . the concern with injustices increases greatly, as does the need to make sense of the events” (p. 1031). Ingroup transgressions, in this regard, should not directly elicit an injustice sense among high-identifiers, as the occurrence of injustice remains confined to the “others’ world.” To be truly concerned with injustice, individuals need to adopt a mind-set transcending their group boundaries (Halabi et al., 2015; Radke et al., 2020). We believe that, relative to high-identifiers, low-identifiers from high-status transgressor groups conform to this notion. This is because their weak ingroup commitment may make them more open to the unjust elements of transgressions, enabling them to perceive events without an ingroup bias (also see Clayton & Opotow, 2003; Doosje et al., 1998). This in turn may stimulate them to exhibit solidarity with victims. Supporting this argument, Teixeira et al. (2023) found that only low-identifiers from high-status groups exhibited unwavering support for protests initiated by low-status groups, regardless of the level of violence involved, when they perceived these protests as a means to dismantle prevailing intergroup disparities. Similarly, we argue that low-identifiers from high-status transgressor groups are more likely than high-identifiers to perceive the events as unjust, and demonstrate solidarity intentions to address this injustice.

Furthermore, Adra et al. (2020) found that perceived injustice drives high-status group members’ outgroup solidarity orientations. We suggest that this link may be even stronger when considering the affective experiences stemming from the injustice (also see Mackie et al., 2000; C. A. Smith & Lazarus, 1993). These affective experiences may include action-oriented negative emotions like anger and outrage (Thomas et al., 2009). These “justice-related” emotions have been found to predict prosocial outgroup intentions in cases of ingroup transgressions (e.g., Iyer et al., 2007). Their role in motivating actions aligns with findings indicating that unfairness perceptions predicted collective action intentions better when coupled with emotional experiences (Agostini & van Zomeren, 2021; H. J. Smith et al., 2012; van Zomeren et al., 2008). Similar to the role of image-related emotions in the image-based solidarity path of high-identifiers, we consider justice-related emotions as the emotional catalysts for the justice-based solidarity path of low-identifiers. In other words, “if the justice motivation and emotional arousal are sufficiently strong, . . . people will be less responsive to other motivational considerations, including self-interested goal acquisition and *impression management*” (Lerner, 2003, p. 396, italics added).

One may question these emotions’ relevance to low-identifiers. In the context of high-status ingroup transgressions, justice-related emotions can be directed toward the ingroup, as a blaming response to group members’ wrongdoings (Thomas et al., 2009). Indeed, these emotions are considered a means of distancing oneself from the targets they were felt toward (see Fischer & Manstead, 2008). Low-identifiers may be more likely than high-identifiers to experience justice-related emotions, as their weaker ingroup loyalty may lead to more distancing from the ingroup when witnessing ingroup transgressions (Radke et al., 2020). Accordingly, we assert that, in comparison to high-identifiers, perceived injustice from transgressions and justice-related emotions constitute the roots of low-identifiers’ solidarity with victimized low-status groups.

## The Current Research

**Figure 1.**
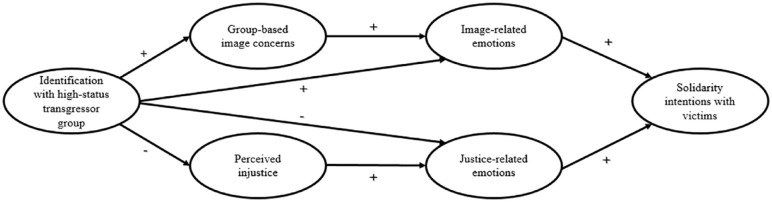
A Model of High-Status Group Solidarity With Victimized Low-Status Groups in the Face of High-Status Group Transgressions.

In summary, we postulated that high-identifiers are more likely than low-identifiers to show solidarity with victimized low-status groups by following the *image-based* solidarity route. Their primary motivation is to repair their group’s image and alleviate feelings of guilt and shame (i.e., image-related emotions) resulting from ingroup transgressions. This approach allows them to address their ingroup’s wrongdoings in line with their group’s interests. On the contrary, low-identifiers are more inclined than high-identifiers to express their solidarity by following the *justice-based* solidarity route. Their primary motivation is to restore perceptions of justice and confront transgressions through heightened anger and outrage (i.e., justice-related emotions). Figure 1 illustrates the proposed theoretical model.

We investigated our model among highly educated individuals by presenting them with fictional information about highly educated individuals (i.e., the high-status transgressor group) displaying blatant prejudice and discrimination against lower-educated individuals (i.e., the low-status victimized group). We conducted three studies using cross-sectional and experimental designs. The first two examined the proposed model in the Dutch context. Study 1 tested the suggested associations among the variables in a correlational manner. In Studies 2 and 3, we experimentally manipulated group-based image concerns and perceived injustice using a priming task. Study 3 aimed to replicate the findings in the British context.

Moreover, in Studies 1 and 2, we explored unique solidarity modes by classifying collective action based on radicalism (i.e., the degree of pursuing fundamental change by defying the existing order through extreme and/or disruptive means; Doosje et al., 2016). We categorized solidarity-based collective actions into nonradical and radical action (also see Jiménez-Moya et al., 2015). Nonradical collective actions operate within the boundaries of the existing social order in less costly and confrontational ways (e.g., signing petitions and lobbying). In contrast, radical collective actions involve more costly and unconventional methods, which can be nonnormative and/or violent (e.g., civil disobedience and institutional disruption). In Study 3, we explicitly formulated our hypotheses based on this distinction. In brief, we examined whether image-related concerns and emotions initiate nonradical solidarity among high-identifiers, and justice-related concerns and emotions initiate both solidarity forms among low-identifiers.

We disclosed all study materials, datasets, and analysis scripts in our research (https://osf.io/pcaku/?view_only=6fd25b5b34b346f59346034679db2a3c). No data were collected for a particular study once the analysis phase had commenced. Please refer to the preregistration forms for full analysis plans (Study 1: https://aspredicted.org/W6H_294; Study 2: https://aspredicted.org/ZKW_6FR, Study 3: https://aspredicted.org/89J_L4R) and Appendix for exploratory analyses. All analyses were conducted in R Version 4.2.3 (R Core Team, 2023).

## Study 1

Study 1 aimed to investigate the relationships between identification^1^ with highly educated individuals, various motives (including perceived injustice, group-based image concerns, and justice- and image-related emotions), and solidarity intentions following the mistreatment of lower-educated individuals. We also investigated whether perceived injustice, group-based image concerns, justice- and image-related emotions mediate the links between identification and solidarity intentions. Our hypotheses were as follows:

Higher levels of group-based image concerns, perceived injustice, and image- and justice-related emotions are associated with solidarity intentions (H1). Identification with highly educated people positively predicts group-based image concerns and image-related emotions (H2a). Identification with highly educated people negatively predicts perceived injustice and justice-related emotions (H2b). Group-based image concerns and image-related emotions sequentially mediate the link between higher levels of identification and solidarity intentions (H3a). Perceived injustice and justice-related emotions sequentially mediate the link between lower levels of identification and solidarity intentions (H3b).

### Method

#### Participants and Design

To assess the adequacy of our sample size for testing serial mediational paths, we conducted a Monte Carlo power analysis for indirect effects (Schoeman et al., 2020) using an online application before data collection. The analysis indicated that 229 participants would be sufficient to achieve a power level of .80 at a 95% confidence interval for moderate intercorrelations between variables (i.e., *r* = .30). This calculation assumed a standard deviation of 1.00, with 1000 replications and 20,000 Monte Carlo draws per replication. In November 2022, we recruited 250 first-year undergraduate psychology students from the University’s participant pool system, offering course credit as compensation. Based on preregistered exclusion criteria, we excluded 20 participants in total (*N_survey completion < 2 mins_* = 3, *N_attention checks_* = 9, *N_comprehension checks_* = 8). The remaining were 230 participants (*N_female_* = 169, *N_male_* = 58, *N_non-binary_* = 3). The participants’ age distribution was as follows: *N_16-19 years_* = 122, *N_20-22 years_* = 91, *N_22-25 years_* = 12, and *N_+25 years_* = 5.

The study had cross-sectional design. Identification with highly educated people was included as a predictor, with group-based image concerns and perceived injustice as primary mediators, image- and justice-related emotions as secondary mediators. Solidarity intentions were examined as outcome variables.

#### Procedure

The study was advertised as a survey investigating the views of University of Groningen (RuG) students regarding lower-educated individuals in the Netherlands. After collecting demographics, participants indicated their level of identification with highly educated individuals. Subsequently, all participants read a fabricated newspaper article detailing discrimination against lower-educated people in the Netherlands. The article featured fictitious study results, including figures illustrating that approximately 75% of RuG students as highly educated people held negative perceptions (e.g., viewing them as incompetent, narrow-minded, unfriendly, and antisocial) about lower-educated people and tended to avoid social interactions with facility personnel on campus. The article concluded by emphasizing the need to address this negativity toward lower-educated individuals. Following this, participants completed various scales assessing group-based image concerns, perceived injustice, image- and justice-related emotions, and solidarity intentions. Finally, participants were thanked and debriefed. The data collection procedure received approval from the University’s Ethical Committee of Psychology.

#### Measures

Unless otherwise specified, all responses range from 1 (*strongly disagree*) to 7 (*strongly agree*). Higher scores for all measurements indicate high levels of correspondent variables.

##### Identification With Highly Educated People

Recognizing threats to the ingroup’s reputation primarily depends on the self-investment component of social identification (e.g., Leach et al., 2008; Teixeira et al., 2020). Also, previous work by Vignoles et al. (2021) addresses the construct validity issues of the self-definition component. We therefore assessed identification with highly educated individuals using 10 items from the self-investment component of Leach et al.’s (2008) social identification scale (e.g., “I am glad to be a highly educated person”; α = .90).

##### Group-Based Image Concerns

Three items from the social-image scale of Teixeira et al. (2020) were adapted to measure how threatening participants consider the transgression to their ingroup’s image. Based on the prefix “RuG students” opinions presented here, items included “will make higher-educated people seem bad to other people in society,” “will damage the reputation of higher-educated people in society,” and “will stain the image of higher-educated people in society” (α = .88).

##### Perceived Injustice

Three adapted items from Tausch et al. (2011) assessed how unjust participants consider the transgression to lower-educated people. Based on the same prefix above, items included “illustrate the ongoing unfair treatment against lower-educated people in society,” “illustrate the ongoing social injustice against lower-educated people in society” and “illustrate the ongoing illegitimate treatment against lower-educated people in society” (α = .90).

##### Image- and Justice-Related Emotions

We assessed emotions toward the newspaper article. Participants indicated their emotions based on the prefix “RuG students” opinions presented here make me feel. The factor analysis (see Supplementary Appendix) revealed a distinction between image- (i.e., “guilty,” “ashamed” and “embarrassed”; α = .84) and justice-related emotions (i.e., “angry,” “outraged,” and “appalled”; α = .87) distinction.

##### Solidarity Intentions

Six items, starting with the prefix “We, as higher-educated people, need to,” were utilized to measure solidarity intentions. The factor analysis (see Supplementary Appendix) revealed a two-factorial structure, distinguishing between nonradical and radical solidarity intentions. Two sets of three items corresponded to nonradical action intentions (i.e., “show solidarity with lower-educated people to improve their living conditions in peaceful ways,” “support non-violent actions to raise awareness about the negativity toward lower-educated people,” “take non-radical actions to help lower-educated people overcome social exclusion toward them”; α = .80) and radical action intentions (i.e., “support radical actions in society to show solidarity with lower-educated people,” “support civil disobedience to draw attention to negativity toward lower-educated people,” “take disruptive actions against those who discriminate lower-educated people”; α = .76). We based our analyses on this distinction.

### Results and Discussion

#### Primary Analyses

Table 1 presents descriptive statistics and intercorrelations among the variables. As hypothesized, perceived injustice and justice-related emotions displayed positive associations with both types of solidarity intentions. Conversely, group-based image concerns and image-related emotions displayed either negative associations with radical action intentions or were not significantly associated with nonradical action intentions. These findings partially supported H1 by demonstrating the expected associations between perceived injustice, justice-related emotions, and solidarity intentions. Furthermore, identification with highly educated people was positively correlated with group-based image concerns and image-related emotions, while negatively correlated with perceived injustice and justice-related emotions. These results fully supported H2a and H2b.

**Table 1.** Descriptive Statistics and Intercorrelations Among Main Variables.

	Main variables	M *(SD)*	1	2	3	4	5	6	7
**1**	Identification with highly educated people	4.62 *(0.97)*							
**2**	Group-based image concerns	4.33 *(1.38)*	.32***						
**3**	Perceived injustice	5.58 *(1.16)*	−.24***	−.24***					
**4**	Image-related emotions	4.25 *(1.55)*	.36***	.27***	.01				
**5**	Justice-related emotions	5.01 *(1.38)*	−.23***	−.05	.54***	.05			
**6**	Nonradical action intentions	5.82 *(1.05)*	−.05	−.03	.41***	.07	.50***		
**7**	Radical action intentions	2.66 *(1.38)*	−.23***	−.21**	.33***	-.15*	.39***	.29***	

* *p* < .05, **p < .01, ***p < .001.

#### Testing the Hypothesized Path Model

H3 posited the sequential mediating roles of both perceived injustice and justice-related emotions, as well as group-based image concerns and image-related emotions, in explaining the link between identification with highly educated people and solidarity intentions. However, due to the absence of significant links between group-based image concerns (see Table 1), image-related emotions, and nonradical action intentions, we fully investigated only the sequential mediational role of perceived injustice and justice-related emotions through a path analysis using “lavaan” package (Rosseel, 2012). In so doing, we employed a model based on intercorrelations among variables where all variables were allowed to correlate with each other, except for restricting associations among group-based image concerns, image-related emotions, and nonradical action intentions.^2^ In addition, we restricted the association between identification and nonradical action intentions, as well as the covariance between image- and justice-related emotions. This model enabled us to accurately examine hypothesized associations while controlling for unhypothesised ones.

**Figure 2.**
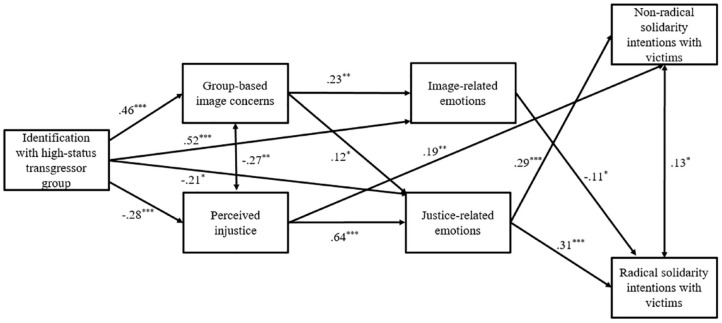
Path Model for Study 1. *Note.* The figure displays only the significant paths, with coefficients represented as standardized estimates. *p < .05, **p < .01, ***p < .001.

We used a standard set of fit indices to assess the model fit: Comparative Fit Index (CFI), Tucker–Lewis Index (TLI), root mean square error of approximation (RMSEA), and standardized root mean square residual (SRMR). The model fit the data well, *χ*^2^(4) = 4.64, *p* = .327, SRMR = 0.03, RMSEA = 0.03, CFI = 0.99. As seen in Figure 2, perceived injustice and justice-related emotions sequentially mediated the associations between identification and both types of solidarity intentions (*β_non-radical_* = −.05, *SE_non-radical_* = .02, 95% CI [−.10, −02.]; *β_radical_* = −.06, *SE_radical_* = .02, 95% CI [−.11, −.02]), while group-based image concerns and image-related emotions did not mediate the association between identification and radical action intentions (*β* = −.01, *SE* = .01, 95% CI [−.04, .01]).

#### Alternative Model Testing

It is also plausible that identification might be linked to solidarity intentions through emotions first and then appraisals. Hence, we tested an alternative model where we changed the order of emotions and appraisals. Although the alternative model fit the data well too, *χ*^2^(4) = 8.21, *p* = .084, SRMR = 0.04, RMSEA = 0.07, CFI = 0.98, the Akaike information criterion (AIC) suggested the empirical superiority of the first model (AIC = 4,381.96) over the alternative (AIC = 4,385.54). Taken together, these findings supported H3b but not H3a.

Study 1 provided correlational evidence that ingroup transgressions evoke different appraisals and emotions among high- and low-identified transgressor group members. Perceived injustice and justice-related emotions drive low-identifiers to engage in solidarity with victimized groups. However, group-based image concerns and image-related emotions, although aroused among high-identifiers, were not linked to solidarity intentions. This is not surprising, especially in the case of radical solidarity, where radical actions might harm the ingroup image and fail to fulfill high-identifiers’ motives (Shuman et al., 2021; Teixeira et al., 2020). Nonetheless, in the case of nonradical solidarity, one can anticipate the action-orienting role of image-related concerns, as demonstrated in previous research (see Shuman et al., 2018). Consequently, we retained this pathway, opting to conduct an experimental investigation of our model.

## Study 2

Treating identification as the predictor, Study 1 provided correlational support for the existence of relatively differential appraisals among low- and high-identifiers. To establish key causal effects based on the findings from Study 1, we manipulated distinct appraisals, namely group-based image concerns and perceived injustice in Study 2. That is, participants were primed with either an image-oriented or justice-oriented mind-set before the same ingroup transgression exposure as in Study 1. In Studies 2 and 3, we treated identification as the moderator in the models to investigate whether the strength of priming effects could differ based on identification. Our hypotheses were as follows:

Participants show stronger image-related emotions in the image prime condition than in the injustice prime condition (H1a). Participants show stronger justice-related emotions in the injustice prime condition than in the image prime condition (H1b). Participants show stronger solidarity intentions in the injustice prime condition than in the image prime condition (H2). Participants show stronger image-related emotions and solidarity intentions in the image prime condition than in the injustice prime condition as their level of identification *increases* (H3a). Participants show stronger justice-related emotions and solidarity intentions in the injustice prime condition than in the image prime condition as their level of identification *decreases* (H3b).

In addition, Study 2 was not well-powered enough to account for the mediational roles of emotions. Therefore, we only explored whether image- and justice-related emotions mediate the effects of the experimental manipulation and identification on solidarity intentions.

### Method

#### Participants and Design

We performed a power analysis for a 2 × 2 (priming: image vs. justice × identification: high vs. low) analysis of variance (ANOVA) design, targeting a small-to-medium interaction effect size (f = .18) using G*Power^3^ (Faul et al., 2009). The analysis revealed that 245 participants provide a statistical power of .80 at an alpha level of <.05. Between January 2023 and February 2023, we recruited 262 first-year undergraduate psychology students from the University’s participant pool system in exchange for course credit. Based on preregistered exclusion criteria, we excluded 12 participants in total (*N_attention checks_* = 7, *N_comprehension checks_* = 5). The remaining were 247 participants (*N_female_* = 179, *N_male_* = 61, *N_non-binary_* = 5, *N_prefer not to say_*= 2). The participants’ age distribution was as follows: *N_16-19 years_* = 110, *N_20-22 years_* = 113, *N_22-25 years_* = 18, and *N_+25 years_* = 6.

The study had between-participant experimental design with two conditions (i.e., the group-based image vs. perceived injustice prime). Identification with highly educated people was the moderator, with image- and justice-related emotions as mediators, and solidarity intentions as the outcomes.

#### Procedure

The study was conducted online using Qualtrics and was advertised as in Study 1. Following demographics, participants reported their level of identification with highly educated people. Subsequently, participants were randomly assigned to experimental conditions. In the experimental manipulation phase, participants completed a task where they imagined themselves, as highly educated individuals, engaging in discussions about education-based disparities in society. In the group-based image prime condition (*N* = 127), they reflected on what other people might think about them, as highly educated individuals, concerning this issue with three sentences. In the perceived injustice prime condition (*N* = 122), participants reflected on the social injustices faced by lower-educated people in society. Following the manipulation, participants read the same ostensible news article as in Study 1. Then, they completed other scales measuring group-based image concerns, perceived injustice, image- and justice-related emotions, and solidarity intentions. Finally, participants were thanked and debriefed. The data collection procedure was approved by the University’s Ethical Committee of Psychology.

### Measures

The scales were identical to the ones used in Study 1. However, this time, group-based image concerns and perceived injustice scales were utilized as manipulation checks. All the scales displayed good reliability scores: identification (α = .91), group-based image concerns (α = .87), perceived injustice (α = .86), image-related emotions (α = .87), justice-related emotions (α = .85), nonradical solidarity (α = .74), and radical solidarity intentions (α = .80).

## Results and Discussion

Descriptive statistics and intercorrelations among the variables across experimental conditions are presented in Supplementary Appendix.

### Manipulation Check

To assess the effectiveness of the experimental manipulation, we conducted independent sample *t*-tests to compare the mean scores of group-based image concerns and perceived injustice between the two experimental conditions. We dummy-coded the experimental conditions: 0 = the group-based image prime, 1 = the perceived injustice prime. Participants in the group-based image prime condition showed significantly higher group-based image concerns (*M* = 4.67, *SD* = 1.34) than those in the perceived injustice prime condition (*M* = 4.05, *SD* = 1.44), *t*(245) = 3.50, *p* < .001. Conversely, participants in the perceived injustice prime condition perceived the transgression as significantly more unjust (*M* = 5.77, *SD* = 0.95) than those in the group-based image prime condition (*M* = 5.28, *SD* = 1.29), *t*(245) = −3.37, *p* < .001. These findings suggested that our experimental manipulation effectively induced distinct appraisals based on the priming condition.

### Testing the Hypotheses

To test our main hypotheses, we conducted moderation models using the PROCESS Macro (Model 1, Hayes, 2017). In each model, we entered the experimental manipulation (dummy-coded) as the predictor, identification (mean-centered; M = 4.79, *SD* = 0.95) as the moderator, image- and justice-related emotions as mediators, and either nonradical or radical action intentions as the outcome. Furthermore, we conducted 5000 bootstrap samples to estimate the standard errors and confidence intervals in these models and other similar ones. All these analysis reports include bootstrapped estimates.

The model with image-related emotions as the outcome yielded significant main effects of the experimental manipulation (*β* = −.44, *SE* = .18, 95% CI [−.80, −.10]), and identification (*β* = .25, *SE* = .11, 95% CI [.05, .46]). These results suggested that the group-based image prime produced more image-related emotions than the perceived justice prime did, which supported H1a. Moreover, high-identifiers (i.e., 1 *SD* above the mean) reported more image-related emotions than low-identifiers (i.e., 1 *SD* below the mean) did. Yet, two-way interaction between them was not significant (*β* = −.02, *SE* = .21, 95% CI [−.43, .38]). These results indicated that the effect of the group-based image prime condition on image-related emotions was not specific solely to high-identifiers; rather, image-related emotions were higher among high-identifiers than low-identifiers independently of the experimental manipulation.

**Figure 3.**
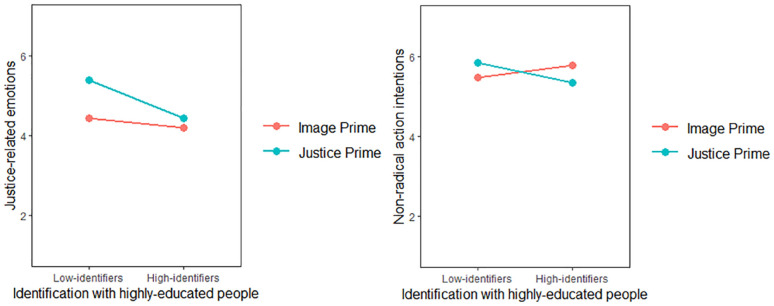
Justice-Related Emotions and Nonradical Action as a Function of Prime and Identification (Study 2).

The model with justice-related emotions as the outcome yielded significant main effects of the experimental manipulation (*β* = .60, *SE* = .17, 95% CI [.26, .92]), and identification, (*B* = −.32, *SE* = .10, 95% CI [−.51, −.14]). Also, two-way interaction between them was significant (*β* = −.38, *SE* = .19, 95% CI [−.75, −.10]). The simple slopes revealed that only low-identifiers in the perceived injustice prime condition reported significantly more justice-related emotions, *β* = .96, *SE* = .24, *t*(243) = 4.07, *p* < .001, see Figure 3. These findings supported H1b and H3b by showing both the main effect of the manipulation and the moderating role of identification on justice-related emotions. They indicated that the perceived injustice priming influenced justice-related emotions specifically among low-identifiers.

The model with nonradical action intentions as the outcome did not reveal significant main effects (*β_manipulation_* = −.03, *SE_manipulation_* = .13, 95% CI [−.29, .24]; *β_identification_* = −.05, *SE_identification_* = .08, 95% CI [−.20, .10]). The absence of the experimental main effect is not in line with H2, which stated that solidarity intentions are stronger in the perceived injustice prime condition than in the group-based image prime condition. However, there was a significant two-way interaction between experimental manipulation and identification (*β* = −.43, *SE* = .15, 95% CI [−.73, −.13]). The simple slopes revealed that low-identifiers in the perceived injustice prime condition, *β* = .38, *SE* = .19, *t*(243) = 2.02, *p* = .044, and high-identifiers (i.e., 1 *SD* above the mean) in the group-based image prime condition, *β* = −.43, *SE* = .19, *t*(243) = −2.32, *p* = .021, reported significantly more nonradical action intentions (see Figure 3). These results indicated that the experimental effects on nonradical solidarity intentions are fully contingent upon identification levels, hence supporting H3a and H3b.

The model with radical action intentions as the outcome yielded significant main effects of the experimental manipulation (*β* = .50, *SE* = .20, 95% CI [.11, .87]), and identification, (*β* = −.31, *SE* = .11, 95% CI [−.54, −.09]). The main effect of the experimental manipulation showed that the perceived injustice priming produced more radical action intentions than the group-based image priming, which partially supported H2. Also, the main effect of identification indicated that low-identifiers reported more radical action intentions than high-identifiers. However, two-way interaction between them was not significant (*β* = −.23, *SE* = .23, 95% CI [−.65, .22]).

### Exploratory Analyses

**Figure 4.**
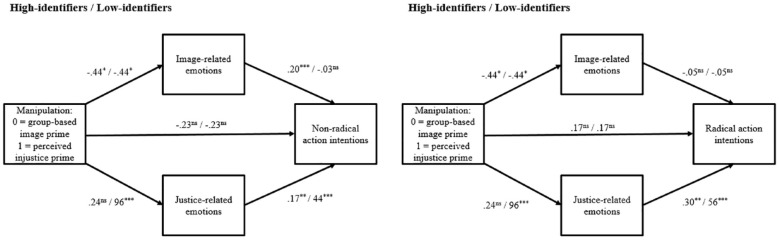
Moderated Parallel Mediation Models With Nonradical and Radical Action Intentions as the Outcomes for High- and Low-Identifiers (Study 2). *Note.* ns = nonsignificant. *p < .05, **p < .01, ***p < .001.

Since our power analysis did not account for the mediational paths, we explored the mediating roles of image- and justice-related emotions on the effects of the experimental manipulation and identification on solidarity intentions, as preregistered. To do this, we conducted moderated mediation models using the PROCESS Macro^4^ (Model 59, Hayes, 2017). In these models,^5^ we entered the experimental manipulation as the predictor, identification as the moderator, image- and justice-related emotions as the parallel mediators, and either non-radical or radical action intentions as the outcome. Figure 4 depicts these models.

In accordance with our theorizing, the model with nonradical action intentions as the outcome revealed that the indirect effect of group-based image priming on nonradical solidarity intentions through image-related emotions was significant among high-identifiers (*β* = −.09, *SE* = .07, 95% CI [−.27, −.02]) but not low-identifiers (*β* = −.01, *SE* = .03, 95% CI [−.04, .08]). Conversely, the indirect effect of perceived injustice priming on nonradical solidarity intentions through justice-related emotions was significant among low-identifiers (*β* = .42, *SE* = .12, 95% CI [.19, .66]) but not high-identifiers (*β* = .04, *SE* = .04, 95% CI [−.04, .14]).

On the contrary, the model with radical action intentions as the outcome revealed that the indirect effect of group-based image priming on radical solidarity intentions through image-related emotions was not significant among either high-identifiers (*β* = .02, *SE* = .06, 95% CI [−.08, .15]) or low-identifiers (*β* = .02, *SE* = .05, 95% CI [−.06, .16]). However, the indirect effect of perceived injustice priming on radical solidarity intentions through justice-related emotions was significant among low-identifiers (*β* = .54, *SE* = .18, 95% CI [.21, .93]) but not high-identifiers (*β* = .07, *SE* = .08, 95% CI [−.06, .25]).

In summary, these results provide experimental evidence regarding the motivational and emotional mechanisms that drive high- and low-identifiers from a high-status group to respond to ingroup transgressions against a low-status group. High-identifiers primed with group-image concerns showed increased image-related emotions, motivating them toward nonradical solidarity intentions. Conversely, low-identifiers primed with injustice concerns reported heightened justice-related emotions, driving them toward both nonradical and radical solidarity intentions. However, these results should be cautiously interpreted, given the study’s limitations, such as inadequate power to examine mediational effects and its reliance on a student sample in the Dutch context. Furthermore, different modes of solidarity intentions in the first two studies were exploratively investigated. We conducted a third study to address these limitations.

## Study 3

Study 3 complemented Study 2 by using the same experimental design with a well-powered sample. It also sought to replicate our findings, extending them to the British context. Drawing upon our previous findings, we explicitly situated our hypotheses on the distinction between nonradical and radical solidarity intentions. Our hypotheses were as follows:

Participants show stronger image-related emotions in the image prime condition than in the injustice prime condition (H1a). Participants show stronger justice-related emotions in the injustice prime condition than in the image prime condition (H1b). Participants show stronger image-related emotions and solidarity intentions in the image prime condition than in the injustice prime condition as their level of identification *increases* (H2a). Participants show stronger justice-related emotions and solidarity intentions in the injustice prime condition than in the image prime condition as their level of identification *decreases* (H2b). Participants show stronger radical action intentions in the injustice prime condition than in the image prime condition as their level of identification *decreases* (H3a). Participants show stronger nonradical action intentions in the image prime condition than in the injustice prime condition as their level of identification *increases* (H3b). Participants show stronger nonradical action intentions in the injustice prime condition than in the image prime condition as their level of identification *decreases* (H3c). Both image- and justice-related emotions mediate the experimental effects on nonradical action intentions (H4a). Only justice-related emotions mediate the experimental effects on radical action intentions (H4b). The mediational role of image-related emotions on the association between the experimental manipulation and nonradical action intentions becomes stronger as the level of identification *increases* (H5a). The mediational role of justice-related emotions on the associations between the experimental manipulation, nonradical and radical action intentions becomes stronger as the level of identification *decreases* (H5b).

### Method

#### Participants and Design

We performed a power analysis based on a 2 × 2 design (priming: image vs. justice × identification: high vs. low), using the smallest interaction effect from Study 2 (f = .1391) via G*Power (Faul et al., 2009). This analysis indicated that 408 participants would provide a statistical power of .80 at an alpha level of <.05. In April 2023, we recruited 419 British adult nationals through Prolific, offering £1.35 per participation. Based on preregistered exclusion criteria, we excluded 10 participants in total (*N_survey completion < 2 mins_* = 4, *N_attention checks_* = 3, *N_comprehension checks_* = 3). The remaining were 409 participants (*N_female_* = 204, *N_male_* = 203, *N_non-binary_* = 1, *N_prefer not to say_*= 1; *M_age_* = 41.05, *SD* = 11.99). The design of the study was the same as in Study 2.

#### Procedure

This online study was advertised as a survey exploring British nationals’ opinions about lower-educated people in the United Kingdom. The procedure and survey flow were the same as in Study 2. However, we adapted the ostensible news article to reflect contextual dynamics, such as referring to highly educated British people in society, a British-named researcher, and a research institution in the United Kingdom. Participants were randomly assigned to the experimental conditions, with 204 participants in the group-based image prime condition and 205 participants in the perceived injustice prime condition. The data collection procedure was approved by the University’s Ethical Committee of Psychology.

#### Measures

The scales were nearly identical to the ones used in Studies 1 and 2. We only replaced the wording “RuG students” with “Highly educated people” in the prefixes. Again, group-based image concerns and perceived injustice scales were utilized as manipulation checks. All the scales displayed good reliability scores: identification (α = .96), group-based image concerns (α = .85), perceived injustice (α = .92), image-related emotions (α = .85), justice-related emotions (α = .90), nonradical solidarity (α = .84), and radical solidarity intentions (α = .87).

### Results and Discussion

Descriptive statistics and intercorrelations among the variables across experimental conditions are presented in Supplementary Appendix.

#### Manipulation Check

We conducted similar independent sample *t*-tests as in Study 2 to examine the effectiveness of the experimental manipulation. Participants in the group-based image prime condition expressed significantly higher group-based image concerns (*M* = 5.49, *SD* = 1.02) than those in the perceived injustice prime condition (*M* = 4.70, *SD* = 1.19), *t*(407) = 3.49, *p* < .001. Conversely, participants in the perceived injustice prime condition perceived the transgression to be significantly more unjust (*M* = 5.84, *SD* = 1.01) than those in the group-based image prime condition (*M* = 5.07, *SD* = 1.53), *t*(407) = −6.04, *p* < .001. These findings suggested that our experimental manipulation effectively induced distinct appraisals based on the priming condition.

#### Testing the Hypotheses

To test the effects of the experimental manipulation and identification with highly educated people on emotions and solidarity intentions, we conducted the same moderation models as in Study 2 by entering the experimental manipulation (dummy-coded) as the predictor, identification (mean-centered; *M* = 4.44, *SD* = 1.31) as the moderator.

The model with image-related emotions as the outcome yielded significant main effects of the experimental manipulation (*β* = −.47, *SE* = .13, 95% CI [−.73, −.22]), and identification (*β* = .33, *SE* = .05, 95% CI [.23, .42]). In line with H1a, the group-based image prime produced more image-related emotions than the perceived justice prime did. Also, high-identifiers reported more image-related emotions than low-identifiers did. Yet, two-way interaction between them was not significant (*β* = −.13, *SE* = .10, 95% CI [−.32, .06]). Replicating Study 2, these results indicated that the effect of the group-based image priming on image-related emotions was not specific solely to high-identifiers; instead, image-related emotions were higher among high-identifiers than low-identifiers independently of the experimental manipulation.

**Figure 5.**
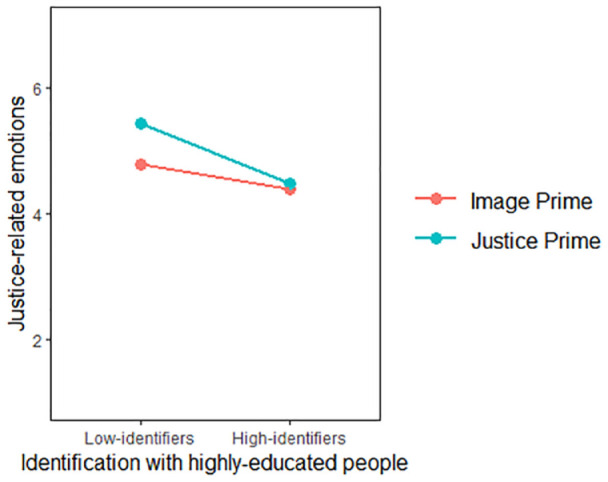
Justice-Related Emotions as a Function of Prime and Identification (Study 3).

The model with justice-related emotions as the outcome yielded significant main effects of the experimental manipulation (*β* = .37, *SE* = .13, 95% CI [.11, .63]), and identification, (*β* = −.26, *SE* = .05, 95% CI [−.35, −.17]). Moreover, two-way interaction between them was significant (*β* = −.21, *SE* = .09, 95% CI [−.39, −.03]). The simple slopes revealed that only low-identifiers in the perceived injustice prime condition reported significantly more justice-related emotions, *β* = .64, *SE* = .18, *t*(405) = 3.55, *p* < .001, see Figure 5. These findings supported H1b and H2b by showing that the effect of the perceived injustice priming on justice-related emotions was specific to low-identifiers.

**Figure 6.**
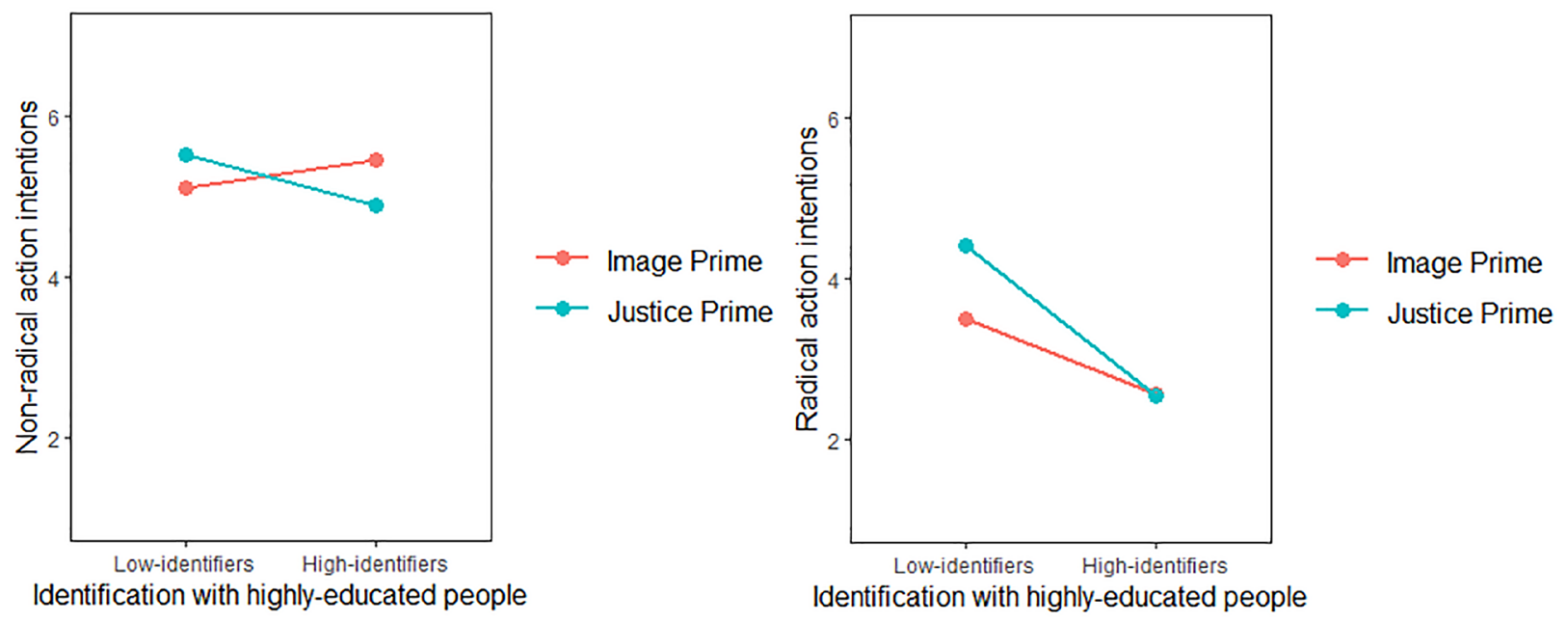
Nonradical and Radical Action Intentions as a Function of Prime and Identification (Study 3).

The model with nonradical action intentions as the outcome did not reveal significant main effects(*β_manipulation_* = −.08, *SE_manipulation_* = .12, 95% CI [−.31, .15]; *β_identification_* = −.06, *SE_identification_* = .04, 95% CI [−.14, .03]). However, there was a significant two-way interaction between the experimental manipulation and identification (*β* = −.37, *SE* = .08, 95% CI [−.53, −.21]). The simple slopes revealed that low-identifiers (i.e., 1 *SD* below the mean) in the perceived injustice prime condition, *β* = .41, *SE* = .16, *t*(405) = 2.49, *p* = .013, and high-identifiers (i.e., 1 *SD* above the mean) in the group-based image prime condition, *β* = −.56, *SE* = .16, *t*(243) = −3.44, *p* < .001, reported significantly more nonradical action intentions (see Figure 6). These findings supported H3b and H3c by showing that the experimental effects on nonradical action intentions are fully contingent upon levels of identification.

The model with radical action intentions as the outcome yielded significant main effects of the experimental manipulation (*β* = .46, *SE* = .15, 95% CI [.16, .74]), identification (*β* = −.54, *SE* = .05, 95% CI [−.64, −.44]), and a two-way interaction between them (*β* = −.36, *SE* = .10, 95% CI [−.55, −.16]). The simple slopes revealed that only low-identifiers in the perceived injustice prime condition reported significantly more radical action intentions, *β* = .93, *SE* = .21, *t*(405) = 4.53, *p* < .001. Figure 6 depicts this interaction. These findings supported H3a by showing that radical action intentions are stronger in the perceived injustice priming than in the group-based image priming as the level of identification decreases.

**Figure 7.**
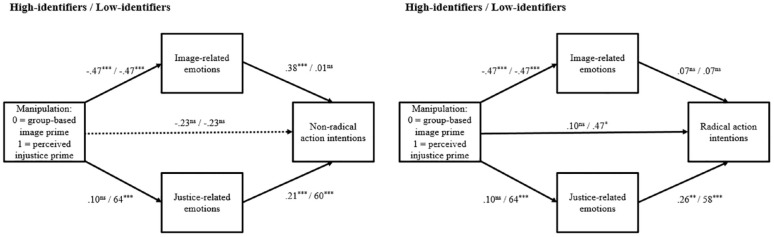
Moderated Parallel Mediation Models With Nonradical and Radical Action Intentions as the Outcomes for High- and Low-Identifiers (Study 3). *Note.* ns = nonsignificant. **p < .01, ***p < .001.

To examine the mediating roles of image- and justice-related emotions on the effects of the experimental manipulation and identification on solidarity intentions, we conducted the same moderated mediation models as in Study 2. Figure 7 summarizes all path coefficients at high and low levels of identification for radical and nonradical solidarity intentions. The model with nonradical action intentions as the outcome revealed that the indirect effect of group-based image priming on nonradical solidarity intentions through image-related emotions was significant among high-identifiers (*β* = −.24, *SE* = .08, 95% CI [−.41, −.09]) but not low-identifiers (*β* = .00, *SE* = .02, 95% CI [−.05, .05]). Conversely, the indirect effect of perceived injustice priming on nonradical solidarity intentions through justice-related emotions was significant among low-identifiers (*β* = .38, *SE* = .11, 95% CI [.18, .61]) but not high-identifiers (*β* = .02, *SE* = .04, 95% CI [−.06, .11]). These findings supported H4a by showing the mediating roles of both image- and justice-related emotions. Also, they provided an initial support for H5a and H5b by illustrating the moderating role of identification on these mediational effects. Note that the effects of the experimental manipulation on nonradical action intentions disappeared after accounting for the mediational paths.

Furthermore, the model with radical action intentions as the outcome revealed that the indirect effect of group-based image priming on radical solidarity intentions through image-related emotions was not significant among either high-identifiers (*β* = .06, *SE* = .05, 95% CI [−.17, .03]) or low-identifiers (*β* = .03, *SE* = .03, 95% CI [−.03, .11]). However, the indirect effect of perceived injustice priming on radical solidarity intentions through justice-related emotions was significant among low-identifiers (*β* = .37, *SE* = .11, 95% CI [.18, .60]) but not high-identifiers (*β* = .03, *SE* = .05, 95% CI [−.07, .15]). These findings supported H4b by showing that only justice-related emotions mediated the experimental effect on radical action intentions. In addition, we posited that the mediational role of image-related emotions on the association between the experimental manipulation and nonradical action intentions becomes stronger as the level of identification increases (H5a) and that holds true for the mediational role of justice-related emotions as the level of identification decreases (H5b). Our findings supported these hypotheses. Moreover, after accounting for the mediational paths, the effects of the experimental manipulation on radical action intentions disappeared only among high-identifiers (*β* = .10, *SE* = .20, 95% CI [−.30, .50]) while it was still significant among low-identifiers (*β* = .47, *SE* = .21, 95% CI [.05, .88]).

Altogether, these results experimentally replicated the findings of Study 2 in a different context with a well-powered sample. Study 3 confirmed that high-identifiers were willing to show solely nonradical solidarity via increased image-related emotions, when they were primed to be concerned about their group image before witnessing the transgression. Conversely, low-identifiers engaged in both nonradical and radical solidarity intentions via justice-related emotions, when they were primed to be concerned about the injustice faced by the low-status group before witnessing the ingroup transgression. Study 3 underscored the significance of different modes of solidarity intentions, particularly for high-identifiers who may strategically pursue preserving their own group image and mitigating their aroused image-related emotions through solidarity expressions.

## General Discussion

Using cross-sectional and experimental data, we examined how appraisals and emotions drive high- and low-identified high-status group members to support lower-status outgroups victimized by the high-status group. The first two studies focused on the Dutch context. Study 1 provided preliminary evidence that, upon witnessing ingroup members transgress against a lower-status group, both high- and low-identifiers evaluated and responded to the situation differently. Compared to high-identifiers, low-identifiers, adopting an outgroup-focused perspective, perceived the transgression as more unjust and experienced more justice-related emotions like anger and outrage. Compared to low-identifiers, high-identifiers, with an ingroup-focused approach, were more concerned about their ingroup’s image and experienced more image-related emotions like guilt and shame. Still, as expected, identification was negatively linked to both nonradical and radical solidarity intentions sequentially through perceived injustice and justice-related emotions. Contrary to our expectations, group-based image concerns and image-related emotions were not linked to action intentions even though there were positive links between them and identification. Nonetheless, Study 2 experimentally demonstrated that priming high- and low-identifiers with an injustice- or image-oriented mindset before witnessing the transgression resulted in distinct emotional responses, driving outgroup solidarity. High-identifiers expressed nonradical solidarity through image-related emotions with image-oriented priming, while low-identifiers expressed both nonradical and radical solidarity through justice-related emotions with injustice-oriented priming. Study 3 replicated these findings in the British context.

Overall, most hypotheses were supported across studies. The hypotheses regarding the justice-based solidarity pathway of low-identifiers were fully supported in all studies. We can thus conclude that the justice-based solidarity pathway reliably explains low-identified transgressor group members’ solidarity. However, findings regarding the image-based solidarity pathway of high-identifiers were less consistent. In the cross-sectional study, group-based image concerns and image-related emotions were not associated with solidarity intentions. In experimental studies, there were no interactive effects of image priming and identification on image-related emotions, but their main effects were significant. Yet, identification fully moderated the associations between image-related emotions and nonradical solidarity. These findings suggest that the image-based solidarity pathway of high-identifiers is less consistent than that of low-identifiers. Note that this pathway initiated outgroup solidarity among high-identifiers compared to low-identifiers in a different transgression context (see Shuman et al., 2018).

### Theoretical and Societal Implications

Our research mostly supported our model’s key hypotheses, and partly replicated and extended prior research. Previous research focused primarily on national transgressions in the United States (Shuman et al., 2018), while our research examined highly educated people’s transgressions toward lower educated people within Dutch and British contexts. Furthermore, this previous research examined how highly identified transgressor group members express solidarity to protect their group’s image. Our study went beyond this by partially replicating these previous findings and adding that low-identifiers were more likely than high-identifiers to feel justice-related emotions when primed with justice concerns before witnessing their ingroup’s transgressions against low-status groups. Compared to high-identifiers, low-identifiers prioritized the outgroup’s suffering over ingroup interests, driving them toward solidarity to restore justice. Essentially, our research contributed by highlighting distinct appraisals and emotions that initiate solidarity among low-identified transgressor group members compared to high-identifiers.

Our findings also highlighted the importance of considering different types of solidarity when examining support from high- and low-identified transgressor group members. Various forms of collective action can be employed in response to group-based transgressions, such as lobbying, sit-ins, property damage, or riots (Becker & Tausch, 2015; Jiménez-Moya et al., 2015). Previous research demonstrated that the actions undertaken by low-status group members to address their plights are influenced by factors such as group efficacy, emotional responses (e.g., anger and disgust), and more (see for reviews, Agostini & van Zomeren, 2021; Becker & Tausch, 2015). Our research indicated that this distinction is also pertinent when high-status transgressor group members engage in solidarity-based collective action. We found that, relative to low-identifiers, high-identifiers, driven by their ingroup’s image concerns and feelings of guilt and shame, favored *nonradical solidarity*. Conversely, relative to high-identifiers, low-identifiers, driven by perceived injustice and feelings of outrage and anger, favored *both nonradical and radical solidarity*.

These findings can be interpreted from high-status group members’ views concerning the transgressor ingroup. Prominent intergroup relations theories (Jost et al., 2004; Sidanius et al., 2004; Tajfel, 1974) posit that high-status group members are primarily driven to maintain their privileged position in societal structures characterized by intergroup disparities. This motivation more strongly influences high-identifiers than low-identifiers, making them hesitate to support radical structural changes (Shuman et al., 2021; Teixeira et al., 2020). Conversely, low-identifiers exhibit this inclination relatively less than high-identifiers due to their lower group investment, leading them to disengage more from their group-based interests (Teixeira et al., 2023). As for ingroup transgressions, high-identifiers, despite being concerned about their groups’s image and experiencing related emotions, tend to express solidarity exclusively through nonradical means, possibly due to their pro-status quo leanings. Conversely, low-identifiers, who disengage more from ingroup interests, may lean toward both nonradical and radical solidarity when they perceive ingroup transgressions as unjust.

Moreover, the outcomes pertaining to the image-based solidarity pathway of high-identifiers should be cautiously interpreted. In Study 1, stronger identification was linked to group-based image concerns and image-related emotions; yet, they were not significantly associated with solidarity intentions. Studies 2 and 3 provided experimental evidence that the activation of group-based image concerns through priming predicted nonradical action intentions among high-identifiers via image-related emotions. Nevertheless, both Studies 2 and 3 observed only the main effects of priming manipulation and identification on image-related emotions. This suggests that the impact of the two variables on image-related emotions operates independently of each other. Image-based solidarity pathway of high-identifiers may require certain conditions (e.g., activation of negative ingroup metastereotypes: van Leeuwen & Täuber, 2012; existence of third-party witnesses: Täuber & van Leeuwen, 2017) to be met to better predict outgroup solidarity. From a social identity perspective, such conditions can pose an identity threat to high-identifiers (Leach et al., 2008), thereby driving outgroup solidarity through image-related emotions. These conditions were absent in the current research, which can explain why we did not observe the interaction between identification and image priming.

Also, the experimental findings of Studies 2 and 3 highlighted the stronger mediating effects of justice-related emotions on action intentions than those of image-related emotions. These findings align with previous research suggesting that confrontational emotions like anger and outrage are more potent predictors of action intentions than aversive emotions like guilt and shame (see Radke et al., 2020). Taken together, our research proposes that the justice-based pathway of low-identifiers is more consistent and a stronger predictor of solidarity than the image-based pathway of high-identifiers.

Moreover, caution is warranted when it comes to interpreting our theoretical rationale regarding high-status transgressors’ outgroup solidarity. We do not necessarily imply that transgressor group members are always strategic, but not moral, in expressing outgroup solidarity. Instead, we introduced which kind of appraisals can be relatively more important for which kind of transgressor group members. From a moral constructivist perspective (Cameron et al., 2015), image and justice concerns can be treated as appraisals (or conceptual knowledge) that drive certain moralistic emotional responses and, consequently, outgroup solidarity. Hence, image and justice concerns can also be seen as moral concerns about harm (Schein & Gray, 2018). With image concerns, the focus is on the harmful effects of ingroup transgressions on the ingroup, whereas with justice concerns, the focus is on the harmful effects on the outgroup. However, the question of to what extent their solidarity is strategically or moralistically driven still requires empirical scrutiny.

In terms of societal implications, our findings underscore that transgressor group members may be driven toward outgroup solidarity for different reasons based on their identification with the transgressor group. In practical terms, activists can make use of these findings. For example, activists may strategically highlight the negative consequences of transgressions for transgressors’ image when aiming to garner solidarity from those highly attached to transgressor groups. Conversely, they may strategically emphasize the negative consequences of transgressions to victims if the goal is to motivate those less attached to transgressor groups for intergroup solidarity. Or, instead of solely focusing on one side, emphasizing the harm of transgressions to both the transgressor and victimized groups could prove more beneficial for fostering favorable intergroup relations among transgressor group members.

### Limitations and Suggestions for Future Research

The current research has some limitations. First, notwithstanding a slight shift in the research context from the Netherlands to the United Kingdom in Study 3, our focus remained on the same education-based transgression. Future research should extend our model to study other transgressions, such as those based on ethnic and gender identities.

Second, while we showed that identification can moderate the justice-based pathway in education-based transgression contexts, this pattern might be more complex in others. For example, in ethnic conflicts, like those seen in the United States, low identification with high-status transgressor groups may not necessarily lead to actions opposing the ingroup’s interests owing to hierarchy-legitimizing ideologies (e.g., color-blindness, see Shuman et al., 2025; political conservatism, see Dai et al., 2021). In addition, low-identifiers are often considered marginal group members who may also respond to ingroup transgressions with indifference or dissent (see Ellemers & Jetten, 2013). Hence, the questions regarding under which circumstances and for which marginal group members lower ingroup identification can foster outgroup solidarity remain crucial. Future research should investigate these nuances by examining the other dynamics of low identification with transgressor groups.

Third, the present research exclusively examined negative emotions that transgressions can evoke. Nevertheless, transgressions could also arise positive emotions (e.g., empathy and sympathy; Radke et al., 2020; Thomas et al., 2009), and identification may play a role in influencing them. Future investigations should give consideration to this aspect as well.

Furthermore, caution is warranted regarding the causality of mediational links through emotions, as emotions were not manipulated in the current research. However, these mediational links were based on previous research and make theoretical sense (Agostini & van Zomeren, 2021; Shuman et al., 2018). It is also important to note that we observed the effects of manipulation on both mediators and outcomes, even though the mediational chains were correlational. Nonetheless, future research should focus on experimentally manipulating emotions to draw conclusions about causal mediational chains.

Finally, one should note that we measured action intentions as behavioral proxies. We employed abstract items to differentiate between nonradical and radical solidarity. Studies 2 and 3 indicated that identification also played a moderating role in the relationship between emotions and action intentions, suggesting that action items could be interpreted differently by high- and low-identifiers. Also, we fall short of concluding what the priming manipulation we used to activate image or justice concerns means for participants. For instance, the image priming might be interpreted as an indirect accusation or an external threat to the ingroup’s image. Which type of interpretation would better predict outgroup solidarity could be an important research question. Future work should place a greater emphasis on measuring more tangible indicators of solidarity and examine different ways of manipulating image and justice concerns.

## Conclusion

In cases of clear-cut high-status group transgressions against lower-status groups, some high-status transgressor group members occasionally show solidarity with low-status groups for their predicaments (Iyer et al., 2007; Shuman et al., 2018). Previous work illustrated that this solidarity is rooted in ingroup-focused appraisals and emotions (i.e., group-based image concerns and image-related emotions). This is a case when individuals identify strongly with the transgressor groups (Shuman et al., 2018). We argued that there are distinct appraisals and emotions (i.e., perceived injustice and justice-related emotions) that explain the solidarity of low-identified transgressor group members. In three studies focusing on education-based discrimination in Dutch and British contexts, we showed that when high-status group members witness ingroup transgressions toward low-status groups, these distinct appraisals and emotions drive their outgroup solidarity differently based on identification with the transgressor group. High-identifiers tended to express solidarity only in nonradical ways via increased group-based image concerns and image-related emotions, while low-identifiers did so in both nonradical and radical ways via increased perceived injustice and justice-related emotions.

## Supplemental Material

sj-docx-1-psp-10.1177_01461672241252871 – Supplemental material for Unraveling Image and Justice Concerns: A Social Identity Account on Appraisals and Emotional Drivers of High-Status Transgressor Group Members’ Solidarity With Low-Status Groups Supplemental material, sj-docx-1-psp-10.1177_01461672241252871 for Unraveling Image and Justice Concerns: A Social Identity Account on Appraisals and Emotional Drivers of High-Status Transgressor Group Members’ Solidarity With Low-Status Groups by Hakan Çakmak, Ernestine H. Gordijn, Yasin Koc and Katherine E. Stroebe in Personality and Social Psychology Bulletin

## Notes

1. Study 1 initially included a disidentification scale (Becker & Tausch, 2014) based on the identification and disidentification distinction. Confirmatory factor analyses (see Supplementary Appendix) did not support this distinction, so we replaced disidentification with low-identification, as preregistered.

2. We also tested the fully saturated model, which produced similar results (see Supplementary Appendix).

3. Sommet et al. (2023) highlighted recent power miscalculations in social psychological research using G*Power, introducing an online app (https://intxpower.com/) to address power issues in 2 × 2 interactions. We were unaware of these advancements during our data collection. Upon discovery, we re-evaluated our power calculation for a fully attenuated interaction, aiming for the same effect size, power, alpha levels, and employing two-tailed testing with contrast. This adjustment required a sample size of 342.

4. In our theoretical model, we posited that differing appraisals and emotions predict solidarity intentions of high- and low-identifiers. Accordingly, the moderated mediation models here were akin to multi-group mediation models where we examine mediational paths for both high- and low-identifiers simultaneously. Model 59 of the PROCESS Macro offers such analyses.

5. The reader should note that, in some models with a continuous moderator (e.g., models 58 and 59), the indirect effect is a nonlinear function of the moderator. The index of moderated mediation is calculated when the indirect effect is a linear function of a single moderator. Therefore, no index is available here (see Hayes, 2017).
