# Supplementary material for: Unraveling Image and Justice Concerns: A Social Identity Account on Appraisals and Emotional Drivers of High-Status Transgressor Group Members’ Solidarity With Low-Status Groups
Source: Pers Soc Psychol Bull. 2024 Jun 18;51(12):2476–93. doi: 10.1177/01461672241252871 (PMC12569109; doi:10.1177/01461672241252871)
Supplement: sj-docx-1-psp-10.1177_01461672241252871 – Supplemental material for Unraveling Image and Justice Concerns: A Social Identity Account on Appraisals and Emotional Drivers of High-Status Transgressor Group Members’ Solidarity With Low-Status Groups [file sj-docx-1-psp-10.1177_01461672241252871.docx]

**Appendix**

**Study 1**

**Confirmatory Factor Analysis on Identification and Disidentification Scales**

In line with what we preregistered, we first explored whether items of identification and disidentification scales fit into the same model via Confirmatory Factor Analysis (CFA). The common method variance was always controlled in this and the following models. This model displayed a poor fit to the data (see Model 1 below). We next explored whether the model with identification and disidentification items being entered as two dimensions shows a better fit. This model also displayed poor fit to the data (see Model 2 below). Following these, we entered identification and disidentification items into separate models, as proposed in the original papers (see Becker & Tausch, 2014; Leach et al., 2008). Specifically, for identification items, we defined ‘solidarity’, ‘satisfaction’, and ‘centrality’ sub-dimensions and allowed them to covariate. This model for identification displayed a very good fit to the data (see Model 3 below). Also, all items loaded very well into correspondent sub-dimensions, ranging from .56 to .86. For disidentification items, we defined ‘detachment’, ‘dissatisfaction’, and ‘dissimilarity’ sub-dimensions and allowed them to covariate. Fit indices were good for this model as well (see Model 4 below). However, item loadings into latent variables indicated that one ‘dissatisfaction’ item (i.e. ‘I wish I had nothing to do with other higher-educated people’) loaded negatively into the correspondent factor (-.36; *p* = .14). Also, two items (i.e. ‘I am unhappy about being a higher-educated person’, ‘Being a higher-educated person gives me a bad feeling’) loaded below .30 into the same factor (.22, *p* < .01; .28, *p* < .05). Consequently, there was just one item for dissatisfaction sub-dimension left. Accordingly, we excluded the dissatisfaction sub-dimension and run the model with only the ‘detachment’ and ‘dissimilarity’ sub-dimensions. The fit indices for this model were good as well (see Model 5 below). However, one detachment item (i.e. ‘I have nothing in common with other higher-educated people’) displayed bad factor loading ( .29, *p* < .001). In the final model, we collapsed the remaining disidentification items (i.e. three from dissimilarity and two from detachment) into one dimension. Yet, this model showed a poor fit to the data (see Model 6 below). Based on these results, we decided to exclude disidentification from further analysis due to its poor fit indices. We, then, replaced it with ‘low-identification’ in hypothesis testing, as we stated in our preregistration form.

**Table S1.**

*CFA Results for identification and disidentification scales (Study 1)*

|  | AIC | χ^2^ | df | p | CFI | TLI | RMSEA | SRMR |
| --- | --- | --- | --- | --- | --- | --- | --- | --- |
| **Model 1** | 13245.027 | 618.898 | 168 | <.001 | .806 | .781 | .108 | .075 |
| **Model 2** | 13300.134 | 676.005 | 169 | <.001 | .782 | .755 | .114 | .080 |
| **Model 3** | 6578.222 | 48.005 | 31 | <.001 | .986 | .979 | .049 | .032 |
| **Model 4** | 6418.814 | 47.360 | 31 | <.001 | .983 | .976 | .048 | .058 |
| **Model 5** | 4615.750 | 33.015 | 13 | <.001 | .971 | .954 | .082 | .069 |
| **Model 6** | 4080.528 | 44.606 | 15 | <.001 | .931 | .885 | .131 | .082 |

**Confirmatory Factor Analysis on Solidarity-based Collective Action Scale**

We next explored the dimensionality of solidarity-based action intentions scale via CFA. The model where we distinguished between non-radical and radical action intentions showed acceptable fit indices, *χ*^2^(8) = 20.83, *p* < .01, SRMR = 0.05, RMSEA = 0.08, CFI = 0.97. With an alternative model, we also entered all solidarity-based action intentions items into one dimension. CFA results demonstrated poor fit indices for this model, *χ*^2^(9) = 161.71, *p* < .001, SRMR = 0.16, RMSEA = 0.27, CFI = 0.64. Moreover, the Akaike information criterion (AIC) suggested the empirical superiority of the first model (AIC = 4463.75) over the alternative model (AIC = 4602.63). Therefore, we concluded that the solidarity-based action intentions scale displays two-dimensional structure (i.e., non-radical vs. radical action intentions), and then we based our main analysis on this distinction in Study 1.

**Exploratory Factor Analysis on Image- and Justice-related Emotions Scales**

We also assessed the factorial structure of the emotion scales. In doing so, we first checked the factorability of the emotion items. Intercorrelations between image- and justice-related emotions were lower than .30 (see Table S2), indicating that these emotions were distinct from each other in the context of ingroup transgressions (see Tabachnick & Fidel 2013). The Kaiser-Meyer-Olkin (KMO) measure of sampling adequacy was .71 (acceptable coefficient is > .60) and the Bartlett’s test of sphericity was significant, *χ*^2^(8) = 695.23, *p* < .001. These indices suggested the suitability of Exploratory Factor Analysis (EFA). Further, we conducted EFA with Maximum Likelihood Factoring (MLF) using oblimin rotation. EFA revealed two factorial solution with high eigenvalues (image-related emotions = 2.20 and justice-related emotions = 2.53). Table S3 summarises the factor loadings. We thus concluded that image- and justice-related emotions displayed distinct constructs in Study 1.

| **Table S2.** | | | | | | | |
| --- | --- | --- | --- | --- | --- | --- | --- |
| *Intercorrelations among emotion items (Study 1)* | | | | | | | |
|  |  | **1** | **2** | **3** | **4** | **5** | **6** |
| **1** | Angry |  |  |  |  |  |  |
| **2** | Outraged | .74^***^ |  |  |  |  |  |
| **3** | Appalled | .65^***^ | .66^***^ |  |  |  |  |
| **4** | Guilty | -.08 | -.19^**^ | -.17^*^ |  |  |  |
| **5** | Ashamed | .19^**^ | .07 | .14^*^ | .54^***^ |  |  |
| **6** | Embarrassed | .20^**^ | .08 | .10 | .56^***^ | .80^***^ |  |
| Notes. ^*^*p* < .05, ^**^*p* < .01, ^***^*p* < .001. | | | | | | | |

**Table S3.**

*Factor loadings of emotion items (Study 1)*

| **Items** | **Factor loadings** | |
| --- | --- | --- |
| ***Justice-related emotions*** |  | |
| Angry | *.86* |  |
| Outraged | *.87* |  |
| Appalled | *.76* |  |
| ***Image-related emotions*** |  | |
| Guilty |  | *.65* |
| Ashamed |  | *.87* |
| Embarrassed |  | *.90* |
| *Note. The loadings lower than .30 were omitted.* | | |

**The roles of political orientation and parental educational background**

Lastly, we explored the moderating role of political orientation in the proposed associations as well as the role of parental educational background as a control variable. Political orientation was assessed using a single 7-point Likert item, with responses ranging from “Extremely Liberal” (1) to “Extremely Right” (7). Parental educational background was evaluated through two 6-point Likert items, measuring the highest educational attainment of both parents, with response options ranging from “No education” (1) to “PhD or a higher degree” (6). Then, we examined the moderating role of political orientation on the sequential mediation of group-based image concerns and image-related emotions, as well as the sequential mediation of perceived injustice and justice-related emotions, on the link between identification with highly-educated individuals and both types of solidarity intentions, respectively. In this analysis, we also incorporated the father's and mother's educational backgrounds as covariates. For this analyses, we utilised PROCESS Macro (Model 92; Hayes 2017) in R. We selected this model to explore whether there is a significant moderating effect of political orientation on any pathway within the proposed model. The model is depicted in Figure S1 below. None of the interaction terms in any of these models reached significance, *ts*(1, 220) < |1.52|, *ps* > 14. We thus concluded that their was no moderating role of political ideology in the proposed associations.

**Figure S1.**
*The model depicting the moderating role of political orientation, including parental educational background as a covariate (Study 1)*


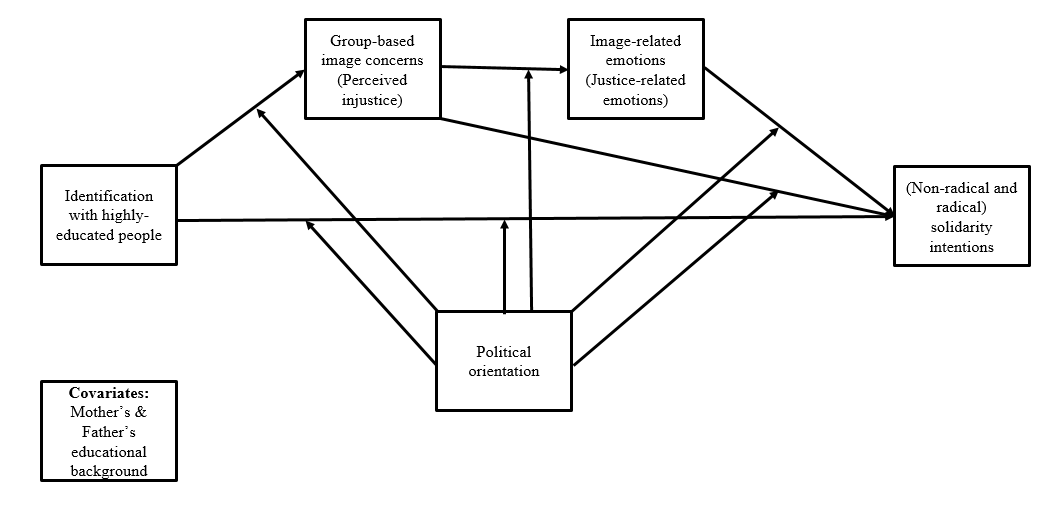


**Figure S2.**
*The significant path model associations in a fully-saturated model*

*
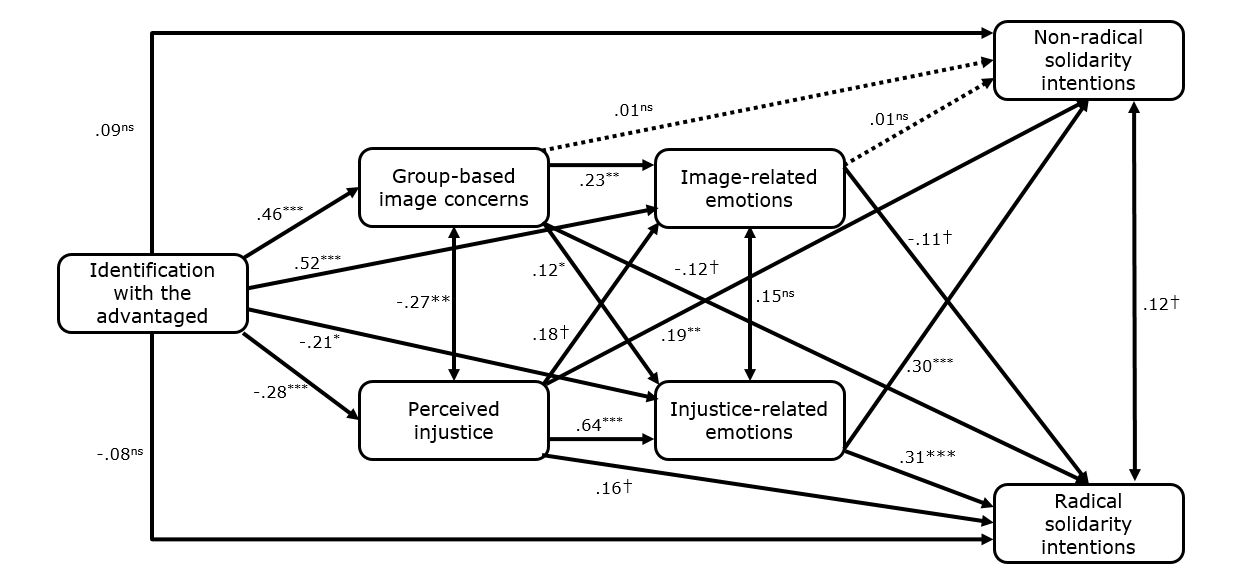
*

**Study 2**

**Table S4**

*Means and standard deviations by condition (Study 2)*

|  | Group-based image prime condition  **(*N* = 125)** | Perceived injustice prime condition  **(*N* = 122)** |
| --- | --- | --- |
| Identification with highly-educated people | 4.81 (0.90) | 4.77 (1.00) |
| Group-based image concerns (Manipulation check) | 4.67 (1.34) | 4.05 (1.44) |
| Perceived injustice (Manipulation check) | 5.28 (1.29) | 5.77 (0.95) |
| Image-related emotions | 4.83 (1.34) | 4.37 (1.53) |
| Justice-related emotions | 4.31 (1.40) | 5.77 (0.95) |
| Non-radical action intentions | 5.64 (0.92) | 5.61 (1.16) |
| Radical action intentions | 3.29 (1.54) | 3.80 (1.58) |
| Emotional coldness* | 2.38 (1.11) | 2.30 (1.10) |
| Reparation attitudes* | 4.84 (1.29) | 4.79 (1.55) |
| Exoneration attitudes* | 2.71 (1.00) | 2.68 (1.10) |
| Political Orientation* | 3.24 (1.17) | 3.21 (1.29) |
| Note. **Variables measured for exploratory purposes.* | | |

| **Table S5.** | | | | | | | |  |  |  |  |  |
| --- | --- | --- | --- | --- | --- | --- | --- | --- | --- | --- | --- | --- |
| *Intercorrelations among variables (Study 2)* | | | | | | | |  |  |  |  |  |
|  |  | **1** | **2** | **3** | **4** | **5** | **6** | **7** | **8** | **9** | **10** | **11** |
| **1** | Identification with highly educated people |  |  |  |  |  |  |  |  |  |  |  |
| **2** | Group-based image concerns | .23^***^ |  |  |  |  |  |  |  |  |  |  |
| **3** | Perceived injustice | -.04 | -.30^***^ |  |  |  |  |  |  |  |  |  |
| **4** | Image-related emotions | .17^**^ | .31^***^ | .05 |  |  |  |  |  |  |  |  |
| **5** | Justice-related emotions | -.24^***^ | -.21^***^ | .55^***^ | .20^**^ |  |  |  |  |  |  |  |
| **6** | Non-radical action intentions | -.07 | -.15^*^ | .33^***^ | .20^**^ | .44^***^ |  |  |  |  |  |  |
| **7** | Radical action intentions | -.19^**^ | -.23^***^ | .39^***^ | -.01 | .41^***^ | .30^***^ |  |  |  |  |  |
| **8** | Emotional coldness | .19^**^ | .20^**^ | -.37^***^ | -.27^***^ | -.55^***^ | -.41^***^ | -.11 |  |  |  |  |
| **9** | Reparation attitudes | -.14^*^ | -.17^**^ | .26^***^ | .21^**^ | .42^***^ | .50^***^ | .31^***^ | -.45^***^ |  |  |  |
| **10** | Exoneration attitudes | .16^*^ | .29^***^ | -.33^***^ | -.15^*^ | -.36^***^ | -.35^***^ | -.10 | .45^***^ | -.45^***^ |  |  |
| **11** | Political orientation | .17^**^ | .25^***^ | -.33^***^ | -.09 | -.37^***^ | -.29^***^ | -.28^***^ | .32^***^ | -.22^***^ | .29^***^ |  |
| Notes. ^*^*p* < .05, ^**^*p* < .01, ^***^*p* < .001. | | | | | | | |  |  |  |  |  |

**Confirmatory Factor Analysis on Solidarity-based Collective Action Scale**

In Study 2, we introduced three negatively-worded items (i.e., “Remind lower-educated people of the reasons why they have lower-status in society [NI1]”, “Protect the well-deserved status we have in society [NI2]”, “Take care that we get what we are entitled to [N3]”) as part of the solidarity measurement. As a first step, we assessed the factorability of the scale items. The intercorrelations among the items revealed that the negatively-worded items exhibited correlations below .30 with the other items (referred to as S1, S2, S3, S4, S5, and S6 in Table S6). This suggests the possibility that these items could constitute a distinct construct separate from the original scale used in Study 1.

| **Table S6.** | | | | | | | |  |  |  |
| --- | --- | --- | --- | --- | --- | --- | --- | --- | --- | --- |
| *Intercorrelations among solidarity items (Study 2)* | | | | | | | |  |  |  |
|  |  | **1** | **2** | **3** | **4** | **5** | **6** | **7** | **8** | **9** |
| **1** | N1 |  |  |  |  |  |  |  |  |  |
| **2** | N2 | .40^***^ |  |  |  |  |  |  |  |  |
| **3** | N3 | .39^***^ | .56^***^ |  |  |  |  |  |  |  |
| **4** | S1 | -.12 | -.01 | -.07 |  |  |  |  |  |  |
| **5** | S2 | -.11 | -.09 | -.20^**^ | .53^***^ |  |  |  |  |  |
| **6** | S3 | -.12 | -.10 | -.05 | .44^***^ | .51^***^ |  |  |  |  |
| **7** | S4 | .02 | -.11 | -.03 | .39^***^ | .19^**^ | .16^*^ |  |  |  |
| **8** | S5 | .09 | .02 | .08 | .26^***^ | .15^*^ | .22^***^ | .58^***^ |  |  |
| **9** | S6 | .11 | .01 | .11 | .24^***^ | .15^*^ | .13^*^ | .53^***^ | .62^***^ |  |
| Notes. ^*^*p* < .05, ^**^*p* < .01, ^***^*p* < .001. | | | | | | | |  |  |  |

Subsequently, we removed these items from further analyses and proceeded to perform CFA using the six solidarity items that were previously used in Study 1. Within this model, our aim was to validate the distinction between non-radical and radical action intentions that we initially identified in Study 1. The model displayed good fit indices, *χ*^2^(7) = 15.94, *p* < .05, SRMR = 0.03, RMSEA = 0.07, CFI = 0.98. Hence, we based our main analysis on this distinction in Study 2 as well.

**Exploratory Factor Analysis on Image- and Justice-related Emotions Scales**

We also examined the factorial structure of the emotion scales. In contrast to Study 1, in Study 2, we included three items aimed at measuring emotional coldness (i.e., unconcerned, indifferent, unmoved) and an additional set of three items measuring positive emotions (i.e., happy, satisfied, proud). As seen in Table S7 below, intercorrelations among emotion items revealed that image- and justice-related emotions could be distinct constructs, as their correlations were lower than .30 (see Tabachnick & Fidell, 2013). However, intercorrelations among other emotion items were generally higher than .30. KMO measure of sampling adequacy was .82 (acceptable coefficient is > .60) and the Bartlett’s test of sphericity was significant, *χ*^2^(66) = 1628.05, *p* < .001. These indices suggested the suitability of EFA. Further, we conducted EFA with MLF using oblimin rotation. EFA revealed four factorial solution with high eigenvalues (image-related emotions = 2.08, justice-related emotions = 4.86, emotional coldness = 1.41, positive emotions = 1.02). Table S8 summarises the factor loadings. Based on these results, we concluded that image- and justice-related emotions, emotional coldness and positive emotions displayed distinct constructs in Study 2.

| **Table S7.** | | | | | | | | | | | | | |
| --- | --- | --- | --- | --- | --- | --- | --- | --- | --- | --- | --- | --- | --- |
| *Intercorrelations among emotion items (Study 2)* | | | | | | | | | | | | | |
|  |  | **1** | **2** | **3** | **4** | **5** | **6** | **7** | **8** | **9** | **10** | **11** | **12** |
| **1** | Angry |  |  |  |  |  |  |  |  |  |  |  |  |
| **2** | Outraged | .70^***^ |  |  |  |  |  |  |  |  |  |  |  |
| **3** | Appalled | .62^***^ | .62^***^ |  |  |  |  |  |  |  |  |  |  |
| **4** | Guilty | .04 | .00 | .04^*^ |  |  |  |  |  |  |  |  |  |
| **5** | Ashamed | .20^**^ | .18^**^ | .28^***^ | .60^***^ |  |  |  |  |  |  |  |  |
| **6** | Embarrassed | .25^***^ | .17^**^ | .25^***^ | .64^***^ | .82^***^ |  |  |  |  |  |  |  |
| **7** | Unconcerned | -.47^***^ | -.44^***^ | -.46^***^ | -.17^**^ | -.38^***^ | -.35^***^ |  |  |  |  |  |  |
| **8** | Indifferent | -.33^***^ | -.40^***^ | -.39^***^ | .02 | -.16^**^ | -.17^**^ | .53^***^ |  |  |  |  |  |
| **9** | Unmoved | -.43^***^ | -.42^***^ | -.46^***^ | -.13^*^ | -.31^***^ | -.31^***^ | .71^***^ | .67^***^ |  |  |  |  |
| **10** | Happy | -.47^***^ | -.45^***^ | -.37^***^ | -.12 | -.16^*^ | -.22^***^ | .46^***^ | .35^***^ | .34^***^ |  |  |  |
| **11** | Satisfied | -.35^***^ | -.35^***^ | -.28^***^ | -.03 | -.12 | -.18^**^ | .47^***^ | .30^***^ | .24^***^ | .67^***^ |  |  |
| **12** | Proud | -.28^***^ | -.26^***^ | -.22^***^ | -.13^*^ | -.20^**^ | -.22^**^ | .45^***^ | .20^**^ | .22^***^ | .59^***^ | .61^***^ |  |
| Notes. ^*^*p* < .05, ^**^*p* < .01, ^***^*p* < .001. | | | | | | | | | | | | | |

**Table S8.**

*Factor loadings of emotion items (Study 1)*

| **Items** | **Factor loadings** | | | |
| --- | --- | --- | --- | --- |
| ***Justice-related emotions*** |  |  |  |  |
| Angry | .82 |  |  |  |
| Outraged | .85 |  |  |  |
| Appalled | .71 |  |  |  |
| ***Image-related emotions*** |  |  |  |  |
| Guilty |  | .73 |  |  |
| Ashamed |  | .88 |  |  |
| Embarrassed |  | .91 |  |  |
| ***Emotional coldness*** |  |  |  |  |
| Unconcerned |  |  | .58 |  |
| Indifferent |  |  | .66 |  |
| Unmoved |  |  | .99 |  |
| ***Positive emotions*** |  |  |  |  |
| Happy |  |  |  | .70 |
| Satisfied |  |  |  | .85 |
| Proud |  |  |  | .76 |
| *Note. The loadings lower than .30 were omitted.* | | | | |

**Exploring whether the hypothesised model predicts reparation and exoneration attitudes**

We further explored whether the hypothesised model predicts reparation and exoneration attitudes. We first conducted moderation models in R using the PROCESS Macro (Model 1; bootstrapped 5000 samples; Hayes, 2017). In these models, we entered the experimental manipulation (dummy-coded) as the predictor, identification (mean-centred; *M* = 4.79, *SD* = 0.95) as the moderator, and reparation and exoneration attitudes as the outcomes, respectively. Starting with the prefix ‘I think, as highly-educated people, we …”, reparation attitudes were assessed four items adapted from previous work (i.e., “need to apologise for all the mistreatment and deprivation that we’ve caused to lower-educated people”, “should apologise to lower-educated people for how we treat them”, “need to repair some of the damage we have caused to lower-educated people”, “should compensate lower-educated people for some of our wrongdoings towards them”; α = .89; Brown et al., 2008; Martinovic et al., 2021). Starting with the prefix “I think …”, three adapted items from Roccas et al. (2006) assessed exoneration attitudes (i.e., “The article exaggerates the discrimination against lower-educated people”, “Even though the article blames us, lower-educated people brought this discrimination upon themselves”, “Even though the article does not mention it, lower-educated people discriminate more against us”; α = .63).

The model with reparation attitudes as the outcome yielded a significant two-way interaction between experimental manipulation and identification (*boot coefficient* = -.48, *boot SE* = .20, 95% CI [-.85, -.10]). However, no main effects reached significance (*boot coefficient_manipulation_* = -.06, *boot SE_manipulation_* = .18, 95% CI [-.41, .28]; *boot coefficient_identification_* = -.19, *boot SE_identification_* = .10, 95% CI [-.38, .01]). The simple slopes revealed that only high-identifiers (i.e., 1 *SD* above the mean) in the group-based image prime condition (*β* = -.52, *SE* = .25, *t*(243) = -2.06, *p* < .05) reported significantly more reparation intentions. These results suggested that our design yielded a similar interactive effect on reparation attitudes as it did on non-radical action intentions. However, this effect was observed only among high-identifiers, unlike in the case of non-radical action intentions.

The model with exoneration attitudes as the outcome yielded a significant two-way interaction between experimental manipulation and identification (*boot coefficient* = .36, *boot SE* = .14, 95% CI [.07, .62]) and a significant main effect of identification *boot coefficient* = .16, *boot SE*= .07, 95% CI [.02, .31]). However, the main effect of the manipulation did not reach significance (*boot coefficient* = -.03, *boot SE* = .13, 95% CI [-.28, .23]). The main effect of identification indicated that high-identifiers were significantly more prone to exonerate the ingroup transgression. Moreover, the simple slopes revealed that only low-identifiers (i.e., 1 *SD* below the mean) in the perceived injustice prime condition (*β* = -.36, *SE* = .19, *t*(243) = -1.94, *p* = .05) reported significantly less exoneration intentions.

Next, we explored the mediating roles of image- and justice-related emotions on the effects of experimental manipulation and identification on reparation and exoneration attitudes. To do this, we conducted moderated mediation models in R using the PROCESS Macro (Model 59, Hayes, 2017). In these models, we entered the experimental manipulation as the predictor, identification (mean-centred) as the moderator, image- and justice-related emotions as the parallel mediators, and reparation and exoneration attitudes as the outcomes, respectively.

The model with reparation attitudes as the outcome revealed that the indirect effect of group-based image priming on reparation attitudes through image-related emotions was not significant among either high-identifiers (*boot coefficient* = -.13, *boot SE* = .10, 95% CI [-.34, .03]) or low-identifiers (*boot coefficient* = .01, *boot SE* = .04, 95% CI [-.07, .11]). However, the indirect effect of perceived injustice priming on reparation attitudes through justice-related emotions was significant among low-identifiers (*boot coefficient* = .45, *boot SE* = .12, 95% CI [.22, .71]) but not high-identifiers (*boot coefficient* = .07, *boot SE* = .07, 95% CI [-.06, .22]). The model with exoneration attitudes as the outcome produced similar results as well. The indirect effect of group-based image priming on exoneration attitudes through image-related emotions was not significant among either high-identifiers (*boot coefficient* = .06, *boot SE* = .05, 95% CI [-.02, .18]) or low-identifiers (*boot coefficient* = .02, *boot SE* = .043 95% CI [-.04, .07]). However, the indirect effect of perceived injustice priming on reparation attitudes through justice-related emotions was significant among low-identifiers (*boot coefficient* = -.30, *boot SE* = .09, 95% CI [-.48, -.13]) but not high-identifiers (*boot coefficient* = -.03, *boot SE* = .04, 95% CI [-.13, .03]). Taken together, these results suggested that the hypothesised model might account only for low-identifiers’ motives for reparation and exoneration.

**Study 3**

**Table S9**

*Means and standard deviations by condition (Study 3)*

|  | Group-based image prime condition  **(*N* = 204)** | Perceived injustice prime condition  **(*N* = 205)** |
| --- | --- | --- |
| Identification with highly-educated people | 4.48 (1.33) | 4.39 (1.29) |
| Group-based image concerns (Manipulation check) | 5.49 (1.02) | 4.70 (1.19) |
| Perceived injustice (Manipulation check) | 5.07 (1.29) | 5.84 (1.01) |
| Image-related emotions | 4.36 (1.38) | 3.86 (1.35) |
| Justice-related emotions | 4.58 (1.35) | 4.98 (1.32) |
| Non-radical action intentions | 5.29 (1.13) | 5.22 (1.25) |
| Radical action intentions | 3.00 (1.52) | 3.51 (1.74) |
| Emotional coldness* | 2.45 (1.07) | 2.48 (1.16) |
| Reparation attitudes* | 4.62 (1.53) | 4.73 (1.56) |
| Justification of discrimination* | 4.74 (1.50) | 4.63 (1.46) |
| Political Orientation* | 3.57 (1.44) | 3.59 (1.25) |
| Note. **Variables measured for exploratory purposes.* | | |

| **Table S10.** | | | | | | | |  |  |  |  |  |
| --- | --- | --- | --- | --- | --- | --- | --- | --- | --- | --- | --- | --- |
| *Intercorrelations among variables (Study 3)* | | | | | | | |  |  |  |  |  |
|  |  | **1** | **2** | **3** | **4** | **5** | **6** | **7** | **8** | **9** | **10** | **11** |
| **1** | Identification with highly educated people |  |  |  |  |  |  |  |  |  |  |  |
| **2** | Group-based image concerns | .12^*^ |  |  |  |  |  |  |  |  |  |  |
| **3** | Perceived injustice | -.03 | -.07 |  |  |  |  |  |  |  |  |  |
| **4** | Image-related emotions | .32^**^ | .28^***^ | .14^**^ |  |  |  |  |  |  |  |  |
| **5** | Justice-related emotions | -.25^***^ | .13^**^ | .56^***^ | .27^***^ |  |  |  |  |  |  |  |
| **6** | Non-radical action intentions | -.05 | .11^*^ | .35^***^ | .38^**^ | .48^***^ |  |  |  |  |  |  |
| **7** | Radical action intentions | -.43^**^ | -.14^**^ | .28^***^ | -.02 | .42^***^ | .45^***^ |  |  |  |  |  |
| **8** | Emotional coldness | .11^*^ | -.19^***^ | -.31^***^ | -.30^***^ | -.57^***^ | -.53^***^ | -.24^***^ |  |  |  |  |
| **9** | Reparation attitudes | -.14^**^ | .02 | .27^***^ | .42^**^ | .44^***^ | .63^***^ | .40^***^ | -.51^***^ |  |  |  |
| **10** | Justification of discrimination | .12^*^ | -.07 | -.14^**^ | .00 | -.30^***^ | -.16^**^ | -.15^**^ | .24^***^ | -.07 |  |  |
| **11** | Political orientation | .14^**^ | .01 | -.17^***^ | -.06 | -.17^***^ | -.21^***^ | -.30^***^ | .11^*^ | -.08 | .18^***^ |  |
| Notes. ^*^*p* < .05, ^**^*p* < .01, ^***^*p* < .001. | | | | | | | |  |  |  |  |  |

**Confirmatory Factor Analysis on Solidarity-based Collective Action Scale**

In Study 3, we used the same items from Study 2. As a first step, we assessed the factorability of the scale items. The intercorrelations among the items revealed that the negatively-worded items exhibited correlations below .30 with the other items (see Table S11). This suggests the possibility that these items could constitute a distinct construct separate from the original scale used in Studies 1 and 2.

| **Table S11.** | | | | | | | |  |  |  |
| --- | --- | --- | --- | --- | --- | --- | --- | --- | --- | --- |
| *Intercorrelations among solidarity items (Study 3)* | | | | | | | |  |  |  |
|  |  | **1** | **2** | **3** | **4** | **5** | **6** | **7** | **8** | **9** |
| **1** | N1 |  |  |  |  |  |  |  |  |  |
| **2** | N2 | .45^***^ |  |  |  |  |  |  |  |  |
| **3** | N3 | .40^***^ | .64^***^ |  |  |  |  |  |  |  |
| **4** | S1 | -.16^**^ | -.21^***^ | -.23^***^ |  |  |  |  |  |  |
| **5** | S2 | -.13^**^ | -.16^**^ | -.14^**^ | .61^***^ |  |  |  |  |  |
| **6** | S3 | -.20^***^ | -.14^**^ | -.12^*^ | .62^***^ | .67^***^ |  |  |  |  |
| **7** | S4 | -.14^**^ | -.18^***^ | -.11^*^ | .44^***^ | .39^***^ | .36^***^ |  |  |  |
| **8** | S5 | .03 | -.15^**^ | .-12^*^ | .27^***^ | .38^***^ | .27^***^ | .61^***^ |  |  |
| **9** | S6 | -.11^*^ | .21^***^ | -.18^***^ | .33^***^ | .39^***^ | .33^***^ | .67^***^ | .81^***^ |  |
| Notes. ^*^*p* < .05, ^**^*p* < .01, ^***^*p* < .001. | | | | | | | |  |  |  |

Subsequently, we removed these items from further analyses and proceeded to perform CFA using the six solidarity items that were previously used in Studies 1 and 2. Within this model, our aim was to validate the distinction between non-radical and radical action intentions that we initially identified. The model displayed good fit indices, *χ*^2^(7) = 24.78, *p* < .001, SRMR = 0.03, RMSEA = 0.08, CFI = 0.99. Therefore, these results corroborated the non-radical vs. radical distinction in solidarity intentions for Study 3 as well.

**Exploratory Factor Analysis on Image- and Justice-related Emotions Scales**

We also examined the factorial structure of the emotion scales. We used the same emotion items from Study 2. As seen in Table S12 below, intercorrelations among emotion items revealed that image- and justice-related emotions could be distinct constructs, as their correlations were lower than .30 (see Tabachnick & Fidell, 2013). However, intercorrelations among other emotion items were generally higher than .30. KMO measure of sampling adequacy was .84 (acceptable coefficient is > .60) and the Bartlett’s test of sphericity was significant, *χ*^2^(66) = 2757.55, *p* < .001. These indices suggested the suitability of EFA. Further, we conducted EFA with MLF using oblimin rotation. EFA revealed four factorial solution with high eigenvalues (image-related emotions = 1.99, justice-related emotions = 5.15, emotional coldness = 1.11, positive emotions = 0.97). Table S13 summarises the factor loadings. Based on these results, we concluded that image- and justice-related emotions, emotional coldness and positive emotions displayed distinct constructs in Study 2.

| **Table S12.** | | | | | | | | | | | | | |
| --- | --- | --- | --- | --- | --- | --- | --- | --- | --- | --- | --- | --- | --- |
| *Intercorrelations among emotion items (Study 3)* | | | | | | | | | | | | | |
|  |  | **1** | **2** | **3** | **4** | **5** | **6** | **7** | **8** | **9** | **10** | **11** | **12** |
| **1** | Angry |  |  |  |  |  |  |  |  |  |  |  |  |
| **2** | Outraged | .80^***^ |  |  |  |  |  |  |  |  |  |  |  |
| **3** | Appalled | .72^***^ | .76^***^ |  |  |  |  |  |  |  |  |  |  |
| **4** | Guilty | .03 | .08 | .03 |  |  |  |  |  |  |  |  |  |
| **5** | Ashamed | .22^**^ | .30^**^ | .34^***^ | .58^***^ |  |  |  |  |  |  |  |  |
| **6** | Embarrassed | .23^***^ | .31^**^ | .38^***^ | .58^***^ | .82^***^ |  |  |  |  |  |  |  |
| **7** | Unconcerned | -.35^***^ | -.42^***^ | -.49^***^ | -.05 | -.25^***^ | -.25^***^ |  |  |  |  |  |  |
| **8** | Indifferent | -.42^***^ | -.48^***^ | -.49^***^ | -.11^*^ | -.32^***^ | -.31^***^ | .62^***^ |  |  |  |  |  |
| **9** | Unmoved | -.43^***^ | -.51^***^ | -.50^***^ | -.11^*^ | -.34^***^ | -.30^***^ | .59^***^ | .72^***^ |  |  |  |  |
| **10** | Happy | -.34^***^ | -.40^***^ | -.38^***^ | .04 | -.17^***^ | -.17^***^ | .38^***^ | .32^***^ | .38^***^ |  |  |  |
| **11** | Satisfied | -.39^***^ | -.39^***^ | -.46^***^ | .06 | -.20^***^ | -.19^***^ | .42^***^ | .41^***^ | .45^***^ | .53^***^ |  |  |
| **12** | Proud | -.36^***^ | -.31^***^ | -.35^***^ | -.07 | -.29^***^ | -.25^***^ | .35^***^ | .39^***^ | .31^***^ | .39^***^ | .54^***^ |  |
| Notes. ^*^*p* < .05, ^**^*p* < .01, ^***^*p* < .001. | | | | | | | | | | | | | |

**Table S13.**

*Factor loadings of emotion items (Study 3)*

| **Items** | **Factor loadings** | | | |
| --- | --- | --- | --- | --- |
| ***Justice-related emotions*** |  |  |  |  |
| Angry | .91 |  |  |  |
| Outraged | .91 |  |  |  |
| Appalled | .71 |  |  |  |
| ***Image-related emotions*** |  |  |  |  |
| Guilty |  | .71 |  |  |
| Ashamed |  | .88 |  |  |
| Embarrassed |  | .89 |  |  |
| ***Emotional coldness*** |  |  |  |  |
| Unconcerned |  |  | .65 |  |
| Indifferent |  |  | .92 |  |
| Unmoved |  |  | .77 |  |
| ***Positive emotions*** |  |  |  |  |
| Happy |  |  |  | .53 |
| Satisfied |  |  |  | .87 |
| Proud |  |  |  | .57 |
| *Note. The loadings lower than .30 were omitted.* | | | | |

**Exploring whether the hypothesised model predicts reparation attitudes**

As in Study 2, we explored whether the hypothesised model predicts reparation attitudes in Study 3. To do this, we first conducted the same moderation model in R using PROCESS (Model 1; bootstrapped 5000 samples; Hayes, 2017). The model yielded a significant two-way interaction between experimental manipulation and identification (*boot coefficient* = -.33, *boot SE* = .11, 95% CI [-.55, -.10]) and the main effect of identification (*boot coefficient* = -.17, *boot SE* = .11, 95% CI [-.28, -.06]). However, the main effect of the manipulation did not reach significance (*boot coefficient* = .09, *boot SE* = .15, 95% CI [-.20, .39]). The main effect of identification indicated that low-identifiers were significantly more prone to reparation, which differed from what we found in Study 2. Moreover, the simple slopes revealed that only low-identifiers in the perceived injustice prime condition (*β* = .52, *SE* = .21, *t*(243) = 2.44, *p* < .05) reported significantly more reparation intentions, which was also inconsistent with what we found in Study 2. Taken together, these results suggested that our hypothesised model did not produce consistent effects of experimental manipulation and identification on reparation attitudes across the two studies.

As in Study 2, we next explored the mediating roles of image- and justice-related emotions on the effects of the experimental manipulation and identification on reparation attitudes using PROCESS (i.e., Model 59; bootstrapped 5000 samples; Hayes, 2017) in R. The moderated mediation model revealed that the indirect effect of group-based image priming on reparation attitudes through image-related emotions was significant among high-identifiers (*boot coefficient* = -.50, *boot SE* = .16, 95% CI [-.82, -.18]) but not low-identifiers (*boot coefficient* = .00, *boot SE* = .03, 95% CI [-.06, .06]). Conversely, the indirect effect of perceived injustice priming on reparation attitudes through justice-related emotions was significant among low-identifiers (*boot coefficient* = .43, *boot SE* = .03, 95% CI [.22, .67]) but not high-identifiers (*boot coefficient* = .01, *boot SE* = .03, 95% CI [-.04, .07]). These findings suggested that the hypothesised model produced similar patterns as those found for non-radical action intentions. Nonetheless, due to the inconsistent findings across Studies 2 and 3, these results should be cautiously interpreted.
